# Muscular Arrangement and Muscle Attachment Sites in the Cervical Region of the American Barn Owl (*Tyto furcata pratincola*)

**DOI:** 10.1371/journal.pone.0134272

**Published:** 2015-07-29

**Authors:** Mark L. L. M. Boumans, Markus Krings, Hermann Wagner

**Affiliations:** Institute of Zoology, RWTH Aachen University, Aachen, Germany; University of Pennsylvania, UNITED STATES

## Abstract

Owls have the largest head rotation capability amongst vertebrates. Anatomical knowledge of the cervical region is needed to understand the mechanics of these extreme head movements. While data on the morphology of the cervical vertebrae of the barn owl have been provided, this study is aimed to provide an extensive description of the muscle arrangement and the attachment sites of the muscles on the owl’s head-neck region. The major cervical muscles were identified by gross dissection of cadavers of the American barn owl (*Tyto furcata pratincola*), and their origin, courses, and insertion were traced. In the head-neck region nine superficial larger cervical muscles of the craniocervical, dorsal and ventral subsystems were selected for analysis, and the muscle attachment sites were illustrated in digital models of the skull and cervical vertebrae of the same species as well as visualised in a two-dimensional sketch. In addition, fibre orientation and lengths of the muscles and the nature (fleshy or tendinous) of the attachment sites were determined. Myological data from this study were combined with osteological data of the same species. This improved the anatomical description of the cervical region of this species. The myological description provided in this study is to our best knowledge the most detailed documentation of the cervical muscles in a strigiform species presented so far. Our results show useful information for researchers in the field of functional anatomy, biomechanical modelling and for evolutionary and comparative studies.

## Introduction

The capability of owls (Aves: Strigiformes) for large head rotation is well known [1]. A functional description of the cervical anatomy is crucial for a mechanistic understanding of the head movements [2]. In addition to the morphology of the vertebrae [3], the vascular system [4], intervertebral cartilage, ligaments, and musculature limit flexibility [3]. In a first attempt to understand the morphological constraints involved in the head movements of owls, the osteology of the cervical vertebrae of the American barn owl (*Tyto furcata pratincola*) was described [5]. In the work presented here we extended this approach by describing the cervical myology of the same species.

A muscle (Latin “musculus”, abbreviated as “M.”, plural “musculi” abbreviated as “Mm.”) which attaches to a cervical vertebra is designated as a cervical muscle, and an overview of the avian cervical musculature based on Baumel et al. (1993) [6] is provided in Table 1. The cervical musculature is the most complex musculature in the avian body [7] with over 200 muscle slips on each side [8]. At the same time it is also the least studied muscle group in birds [9]. The cervical muscles may be classified in four regions based on their relative positions and attachment sites: 1) Mm. craniocervicales, 2) Mm. cervicales dorsales, 3) Mm. cervicales laterales and 4) Mm. cervicales ventrales [6]. The Mm. craniocervicales are continuous with the muscles from the other regions. The Mm. craniocervicales insert on the head and position the head relative to the neck; the muscles from the other three regions position the neck relative to the trunk [6]. The insertion sites of the Mm. craniocervicales surround the foramen magnum. The cervical muscles vary in size: from small intervertebral muscles to long muscles extending over the entire neck. In this first approach to describe the myology of an owl we selected the larger and more superficially located muscles. We reasoned that these muscles are more important for the gross flexibility and head positioning than the smaller muscles because they shorten over greater distances, and cause greater displacements [8].

**Table 1:** Overview of cervical musculature.

Region	Muscle^1^	Part
**1. Mm. craniocervicales**	**M. biventer cervicis** ^**2**^	
	**M. complexus**	
	**M. splenius capitis**	**Pars medialis**
		**Pars lateralis**
	**M. rectus capitis lateralis**	
	**M. rectus capitis dorsalis**	
	**M. rectus capitis ventralis**	**Pars medialis**
		**Pars lateralis**
**2. Mm. cervicales dorsales**	M. longissimus dorsi	
	M. ascendens cervicalis	
	**M. longus colli dorsalis**	**Pars cranialis**
		**Pars caudalis**
		**Pars profunda**
		Pars thoracica
	**M. interspinalis**	
**3. Mm. cervicales laterals**	Mm. intertransversarii	
	Mm. inclusi	
	M. flexor colli lateralis/medialis	
**4. Mm. cervicales ventrales**	**M. longus colli ventralis**	Pars cranialis
		**Pars caudalis**
	M. scalenus	

^1)^ Muscles as described in Handbook of avian anatomy: Nomina Anatomica Avium [6].

^2)^ Muscles selected for description in this work in bold letters.

Gross dissection is a common method for gaining anatomical information [10]. For example, Boas (1929) provided a wealth of anatomical data for the craniocervical region of 34 bird species [11]. He mentioned four owl species, but only the myology of the tawny owl (*Strix aluco*, formerly *Syrnium aluco*) was described in more detail. The description of the tawny owl (*Strix aluco*) by Boas (1929) is to our best knowledge the most detailed description of the cervical myology of an owl species. Masino and Knudsen (1990) mention that there are at least 31 distinct muscle pairs in the neck region of the barn owl (*Tyto alba*) but did not describe them [12]. Kuroda (1962) also studied the avian cervical myology and described the myology of nineteen species within eleven orders [7]. Further descriptions of avian cervical muscles exists from gross dissection for several other species: Shufeldt (1890): raven (*Corvus corax sinuatus*) [13]; Burk (1893): pigeon (*Columba livia*) [14]; Fisher & Goodman (1955): whooping crane (*Grus americana*) [15]; Zusi (1962): black skimmer (*Rynchops nigra*) [16]; Zusi et al. (1969): pied-billed grebes (*Podilymbus* spp.) [17]; Ghetie (1976) and Zweers et al. (1987): chicken (*Gallus gallus domesticus*) [2,18]; Ghetie (1976): turkey (*Meleagris gallopavo*), goose (*Anser anser domesticus*), domestic duck (*Anas domestica*), helmeted guineafowl (*Numida meleagris*) [18]; Jenni (1981): great spotted woodpecker (*Dendrocopos major*) and middle spotted woodpecker (*Dendrocopos medius*) [19]; Zusi & Bentz (1984): several hummingbirds (Trochilidae) [20]; Zusi (1985): noisy scrub-bird (*Atrichornis clamosus*) and superb lyrebird (*Menura novahollandiae*) [21]; Landolt & Zweers (1985): mallard (*Anas platyrhynchos*) [22]. Lautenschlager et al. (2013) were the first to describe avian cervical myology by combining iodine staining with microcomputed tomography [10]. These authors used the common buzzard (*Buteo buteo*) as research material [10]. Tshuihiji (2005, 2007) and Snively and Russell (2007) described and reviewed general avian cervical myology, including many additional species [23–25].

In this study we describe the cervical myology of *T*. *f*. *pratincola* by gross dissection. We investigate the arrangement of selected muscles and their attachment sites. In addition, we want to find out how the regionalisation of the neck as defined by osteology [5] is observable in the myology. *Tyto furcata pratincola* was chosen, because much is known regarding its general behaviour, including head movement behaviour [26], but above all because the morphology of the cervical vertebrae was recently described and models of these vertebrae are available [5]. In addition a regionalisation of the neck based on osteological parameters was described: the first region consists of C1 only followed by two regions with both three vertebrae each (C2-C4 and C5-C7 respectively) [5]. More caudal regions are defined as C8-C9, C10-C12 and C13-C14 respectively [5]. The results of this study supplement the current knowledge according to avian cervical myology, and can function as an anatomical guide for future research. In addition we try to suggest the functional role of the muscles in the natural behaviour of the animals based on our anatomical data and data from other species found in literature.

## Materials and Methods

### Animals

Data from this study were collected from five carcasses of the American barn owl (*Tyto furcata pratincola*, formerly *Tyto alba pratincola*) (Aves: Strigiformes: Tytonidae); three males (two, three and thirteen year old) and two females (both one year old). These individuals originated from the breeding colony of the Institute of Biology II at RWTH Aachen University, Aachen, Germany. The animals had died of natural causes or were perfused for other studies. Thus, no animal was killed for the purposes of this study. No permit is necessary according to European and German law for such a study.

### Dissection

The cervical muscles of *T*. *f*. *pratincola* were examined by gross dissection. The cervical region is defined here as that part of the vertebral column that starts cranially with the first cervical vertebra (the atlas) and ends with those vertebrae that have false ribs (costae incompletae [6]), i.e., ribs that do not attach to the sternum [27]. The cervical region of *T*. *f*. *pratincola* contains fourteen vertebrae [5].

The freshly frozen carcasses were thawed at room temperature. The craniocervical region was separated from the skinned body by cutting the vertebral column at approximately the third thoracic vertebra. Trachea and oesophagus were removed whereas bones, muscles and ligaments were left intact. This separated part of the cervical column will be called “preparation” in the following. The preparation was fixed overnight in 70% ethanol [9] at room temperature and was used for dissection and examination the next day. The sheath of fascia surrounding the muscles was removed with blunt tweezers which enabled the separation of muscles and muscle slips. Dissection was facilitated by a stereomicroscope. During dissection, the preparation was frequently wetted with cold tap water to prevent desiccation. Between subsequent examinations the preparation was stored in 70% ethanol at room temperature.

The vertebra of muscle attachment was determined by palpation and/or by exposure of all previous vertebrae. The vertebrae and muscle attachment sites were documented by photography (Nikon, D70 and Sony, DSC-W35) and schematic drawings. The length of the muscles was measured with a ruler. The termini of the measurement depended on the muscle and are described in more detail in the results section. Three-dimensional models of the vertebrae of *T*. *f*. *pratincola* [5] were used as a reference guide to document the muscle attachment sites. Muscle-bone separation was done with a scalpel.

### Nomenclature

For the anatomical terms throughout this paper we follow Baumel et al. (1993) and refer to a bird standing erect with a fully extended neck, ignoring its natural S-shape [6]. Cervical vertebrae will be abbreviated as ‘C’ followed by its consecutive number [5,28,29]. The first cervical vertebra, C1, is the atlas, followed by C2, the axis/epistropheus, and so on. The last cervical vertebra in *T*. *f*. *pratincola* is C14 [5] which is attached to the first thoracic vertebra (T1). We followed previous authors using the term “origin” of a muscle for the caudal attachment site, and the term “insertion” for the more cranial attachment site [16,22]. Note that the numbering of the vertebrae starts cranially running caudally, whereas the course of the muscles will be discussed from the caudal origin point to the cranial insertion point. The tissue compositions of aponeuroses and tendons are the same, but an aponeurosis has a flat cross section whereas that of a tendon is more oval-shaped [22].

In mathematics and physics three-dimensional coordinate systems are used to divide movements in three rotational and three translational movements. The rotational movements are “yaw” about the vertical axis (z-axis), “pitch” about the transverse axis (x-axis), and “roll” about the longitudinal axis (y-axis) [30]. We call these terms “physical” terms. In biological studies, however, other terms are used that combine the rotations and translations mentioned above to some degree. Such terms are dorsal flexion, ventral flexion, retraction, lateral flexion, rotation [22], extension and protraction [25]. We call these terms “biological” terms. In the following we will preferably use the biological terms, even if they are less precisely defined than the physical terms, due to the fact that movements are not closely related to a single axis. For clarity, the use of abbreviations was avoided as much as possible throughout this work.

## Results and Muscle Specific Discussion

Nine muscles, from which some could be subdivided, were selected for identification: M. complexus, M. biventer cervicis, M. splenius capitis (pars medialis and pars lateralis), M. rectus capitis lateralis, M. rectus capitis ventralis (pars medialis and pars lateralis), M. rectus capitis dorsalis, M. longus colli dorsalis (pars cranialis, pars caudalis and pars profunda), M. interspinalis and M. longus colli ventralis. The following descriptions are based on the dissection of the craniocervical region of five owls from which the results were pooled. In our preparations the only variation found in muscle attachment sites between and/or within specimens, was in the M. complexus (see below). However, we like to mention that not all muscles were examined in all preparations, due to the quality of the preparation and the dissection. The frequency of muscle examination in the different specimens is given in Table 2.

**Table 2:** Overview of frequency of muscle examination in the preparations. Preparation 1: male, three years old, fresh and 70% ethanol. Preparation 2: female, one year old, perfused with 4% paraformaldehyde. Preparation 3: male, thirteen years old, fresh and 70% ethanol. Preparation 4: female, one year old, 70% ethanol. Preparation 5: male, two years old, 70% ethanol. “0” means not examined, “1” means examined.

		Preparation				
		1	2	3	4	5
**Musculus**	Complexus	1	1	1	1	1
	Biventer cervicis	1	1	1	1	1
	Splenius capitis	1	1	1	1	1
	Rectus capitis lateralis	1	1	1	1	1
	Rectus capitis ventralis	1	0	1	1	1
	Rectus capitis dorsalis	0	0	1	1	1
	Longus colli dorsalis, pars cranialis	1	0	1	1	1
	Longus colli dorsalis, pars profunda	0	0	0	0	1
	Longus colli dorsalis, pars caudalis	1	1	0	0	1
	Interspinalis	0	0	0	0	1
	Longus colli ventralis, pars caudalis	1	0	0	0	0

The cervical musculature is composed of several layers of muscles which is surrounded and interconnected by fascia. The individual muscle slips could be separated by careful removal of the fascia. Both fleshy and tendinous muscle attachment sites were found. We identified cervical muscles originating from cervical vertebrae and thoracic vertebrae. The insertions were located on cervical vertebrae and the cranium. All muscles were organised bilaterally, thus present in both the left (sinister) and right (dexter) side of the neck. Several muscles originated from the aponeurosis notarii, a stack of aponeuroses which is located dorsally of the last cervical vertebrae and first thoracic vertebrae [6]. In Table 3 the results of the identified muscles are organised and represented in the four regions of the cervical muscles as described earlier [6].

**Table 3:** Overview of identified muscles in *T*. *f*. *pratincola* with origin and insertion.

Muscle	Origin	Insertion
M. complexus	Diapohyseal processus of processus transversus of C4, C5 and C6	Os supraoccipitale
M. biventer cervicis	Aponeurosis notarii located above C14	Os supraoccipitale
M. splenius capitis, pars lateralis	Processus spinosus of C2	Os supraoccipitale
M. splenius capitis, pars medialis	Dorsal side of arcus vertebrae of C1 and processus spinosus of C2	Os supraoccipitale
M. rectus capitis lateralis	Processus ventralis of C3-C5	Os supraoccipitale
M. rectus capitis ventralis	Processus ventralis of C1-C5	Os basioccipitale
M. rectus capitis dorsalis	Lateral processus of C2-C5	Os basioccipitale
M. longus colli dorsalis, pars caudalis	Aponeuris notarii located above C14 and processus spinosus of C13	Torus dorsalis of C2 and processus transversus of C6-C10
M. longus colli dorsalis, pars cranialis	Processus spinosus of C3-C7	Tendo axialis, which is attached to torus dorsalis of C2
M. longus colli dorsalis, pars profunda	Processus spinosus of C7-C12	Processus transversus of C5-C8
M. interspinalis	Processus spinosus of C3	Processus spinosus of C2
M. longus colli ventralis	Processus ventralis of T2	Processus transversus of C3–C10

### Cervical muscles region 1: Mm. craniocervicales

#### M. complexus

**Fig 1:**
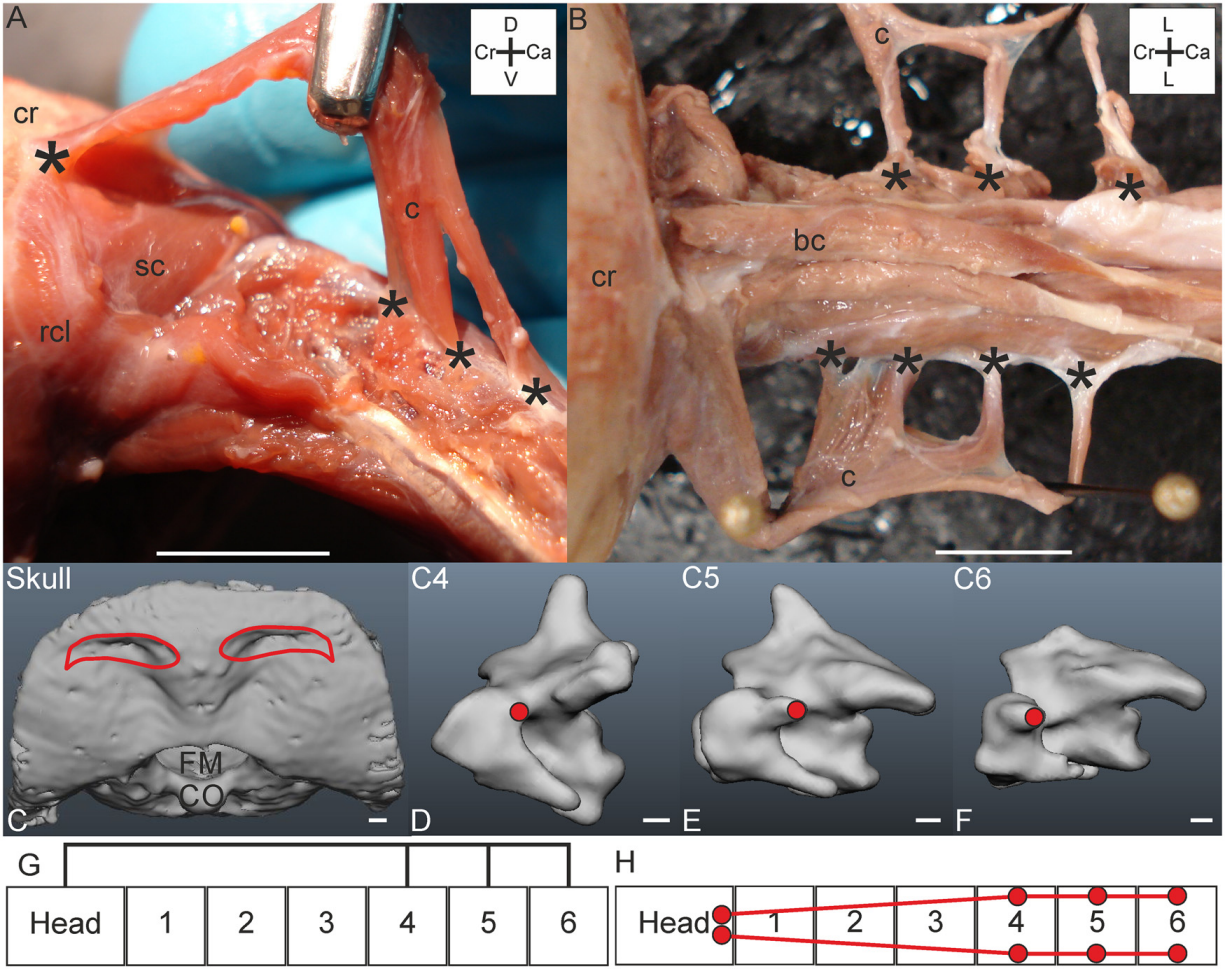
M. complexus. A) Dorsolateral view of M. complexus sinister (c) held up with tweezers; fleshy insertion site on the cranium (cr) and originating muscle slips from cervical vertebrae C4-C6 are indicated by asterisks. The M. splenius capitis (sc) and M. rectus capitis lateralis (rcl) are indicated as a reference. The coordinate system indicates dorsal (D), caudal (Ca), ventral (V) and cranial (Cr). Scale bar represents one centimetre. B) Dorsal view on both M. complexus sinister and dexter (c) showing variation between the sides. The insertion of the M. complexus dexter was separated from the cranium (cr). Individual slips (attaching to C4-C6) are indicated by asterisks. The M. complexus sinister has an extra slip originating from C3. The coordinate system indicates lateral (L), caudal (Ca) and cranial (Cr). Scale bar represents one centimetre. C) Insertion site of M. complexus indicated with red line in three-dimensional model of a part of the skull from dorsal view. Foramen magnum (FM) and condylus occipitalis (CO) are indicated. Scale bar represents one millimetre (adapted from [5]). D-F) Origins of the M. complexus indicated with red circles in the three-dimensional models of the vertebrae of *T*. *f*. *pratincola*; vertebrae (C4-C6) from lateral left view, cranial is to the left. Scale bars in D-F represent one millimetre (adapted from [5]). G) Connection diagram from lateral view of M. complexus in *T*. *f*. *pratincola*; origin and insertion sites are connected with lines representing the muscle slips. H) Connection diagram from dorsal view of M. complexus in which the muscle attachment sites are indicated with red circles and are interconnected by a line representing the muscle.

*Muscle characteristics*: The M. complexus of *T*. *f*. *pratincola* (Fig 1) is a large muscle, approximately four centimetres in length, measured from the most caudal origin to the most cranial insertion point. It is relatively flat and has parallel oriented fibres. The three fleshy slips originate from C4, C5 and C6 (Fig 1A and 1D–1F). In one specimen (specimen 3) an additional attachment site to C3 was found, but only at one side (Fig 1B). The attachment to C3 was clearly not the remnant of another attachment but a case of intraspecific variety which is a regularly documented phenomenon in avian craniocervical myology (see General discussion). The separated slips fuse at the height of the previous (more cranially located) vertebra (Fig 1A and 1B). The muscle then runs cranially and inserts at the os supraoccipitale (Fig 1C). The schemes in Fig 1G and 1H show the connection diagrams from lateral view and dorsal view, respectively.

*Origin*: The three fleshy slips originate tendinously at the diapophyseal processes of the transverse processes of C4, C5 and C6 (Fig 1D–1F).

*Insertion*: The insertion of the M. complexus is the most dorsal attachment site at the os supraoccipitale, near to the craniocaudal axis (Fig 1C). The attachment site is broad and flat, touching the crista nuchalis transversa. The M. complexus covers several other muscles as will become apparent in the following descriptions. The M. complexus dexter and M. complexus sinister do not touch each other medially at the insertion point, but fuse more caudally. There is, thus, a small area on the skull, between the insertion points of the M. complexus dexter and M. complexus sinister which does not serve as muscle attachment site. This area is located a bit higher in relation to the sunken area which serve as muscle attachment site.

*Comparison*: A M. complexus originating from C4, C5 and C6 was also found in the raven (*Corvus corax sinuatus*) [13], the pigeon (*Columba livia*) [14], the domestic fowl (*Gallus gallus*) [31], the huia (*Heteralocha acutirostris*) [27] and in both the great spotted woodpecker (*Dendrocopos major*) and middle spotted woodpecker (*Dendrocopos medius*) [19]. The M. complexus in the tawny owl (*Strix aluco*) and the white-tailed eagle (*Haliaeetus albicilla*) has four slips originating from the processus dorsalis of C3, processus costalis of C4, processus costalis of C5, and tuberculum ansae of C6 [11], respectively. The arrangement is similar as in *T*. *f*. *pratincola*. The M. complexus of *T*. *f*. *pratincola* did not originate from the torus dorsalis (epipophysis) of any cranial cervical vertebra, as occurs in some species [23,25]. A separation of the muscle by tendinous intersections as documented, for example, in the pied-billed grebe (*Podilymbus podiceps*) [17] was not observed in *T*. *f*. *pratincola*.

*Specific discussion*: From electromyographic studies in the chicken (*Gallus gallus domesticus*) it was found that the M. complexus is involved in lateroflexion, dorsiflexion and rolling of the head [32]. The arrangement of the muscle in *T*. *f*. *pratincola* is suited to cause the same movements. The M. complexus originates from osteological region 2 (C4) and region 3 (C5 and C6) as defined by Krings et al. (2014) [5]. This muscle may be involved in minor lateroflexion but may also underlie large rotational capacity in the most cranial vertebrae. The existence of the slips is also related to the increase in the zygapophyseal protrusion and to the occurrence of arterial canals [5]. Whether the correlation with the occurrence of the arterial canal has functional relevance remains unclear.

#### M. biventer cervicis

**Fig 2:**
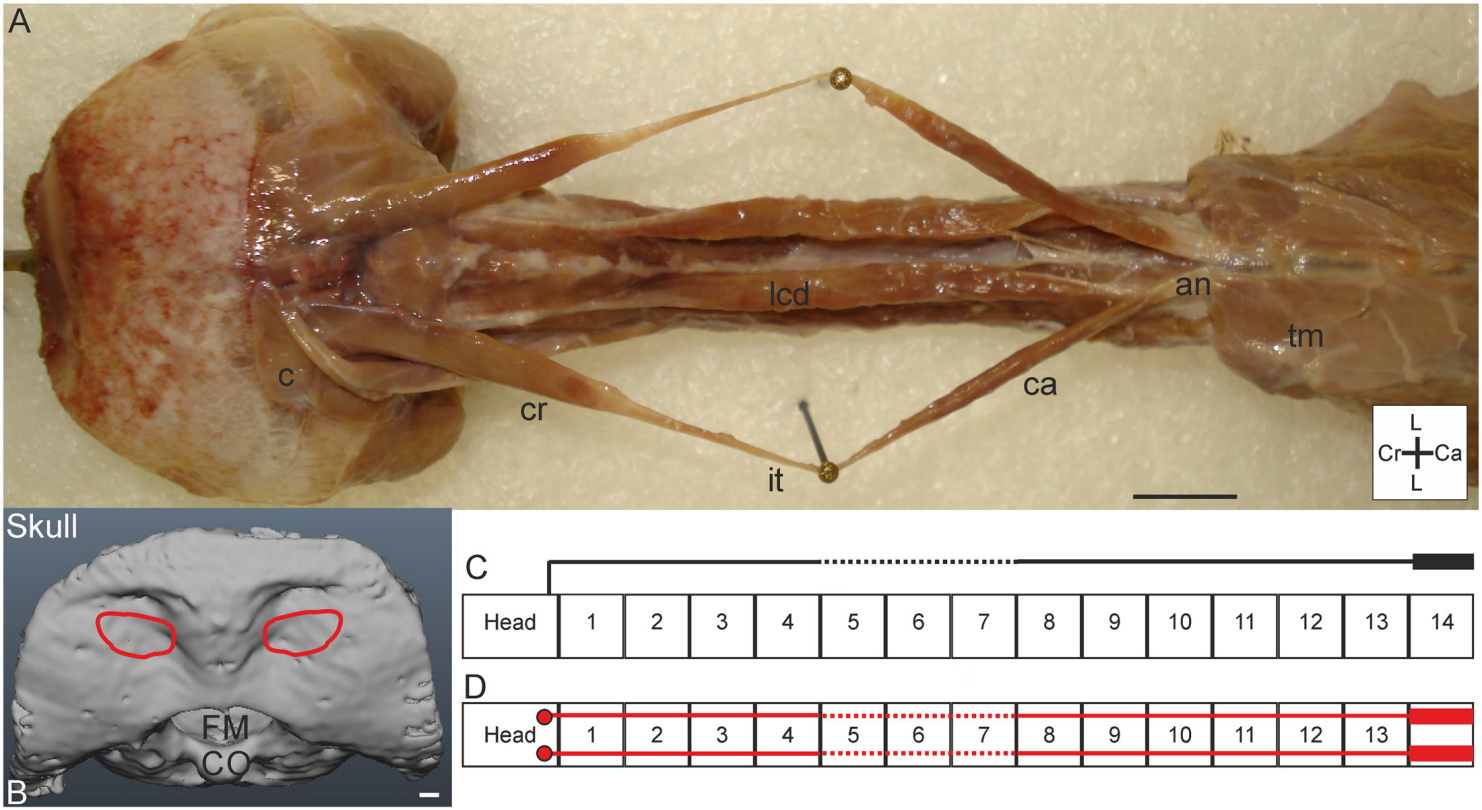
M. biventer cervicis. A) Dorsal view on both M. biventer cervicis sinister and dexter. The two sides are placed apart by needles, but *in situ* located dorsally from the M. longus colli dorsalis (lcd). M. biventer cervicis originates from the dorsocaudally located aponeurosis notarii (an), which is partly covered by thoracic muscles (tm). The cranial (cr) and caudal (ca) bellies are connected by the intersectio tendinea (it). M. biventer cervicis inserts at the cranium, ventral from the insertion of the M. complexus (c). Coordinate system indicates lateral (L), caudal (Ca) and cranial (Cr). Scale bar represents one centimetre. B) Insertion site of the M. biventer cervicis on the skull (red line) as seen from dorsal (D) view. Foramen magnum (FM) and condylus occipitalis (CO) are indicated in the skull. Scale bar represents one millimetre (adapted from [5]). C) Connection diagram from lateral view of M. biventer cervicis in *T*. *f*. *pratincola*; origin and insertion sites are connected with lines representing the muscle slips; broken line represents intersectio tendinea. The heavy line above C14 represents aponeurosis notarii. D) Connection diagram from dorsal view of M. biventer cervicis, in which the muscle attachment sites are indicated with red circles and are interconnected by a line representing the muscle and broken lines indicating intersectio tendinea. The heavy line above C14 represent the aponeurosis notarii.

*Muscle characteristics*: The M. biventer cervicis is relatively long and thin (Fig 2A). It is the only muscle that connects the cranium with the notarium (a unit of fused thoracic vertebrae) [6]. In *T*. *f*. *pratincola* it originates from the aponeurosis notarii (which is located dorsally from C14 and then runs caudally) and inserts at the os supraoccipitale (Fig 2A), spanning a length of more than eight centimetres (Fig 2A). The aponeurosis which gives rise to the M. biventer cervicis even runs more caudally, dorsally from the thoracic vertebrae. The M. biventer cervicis in *T*. *f*. *pratincola* has two parallel fibred fleshy bellies interconnected by a tendon (intersectio tendinea). The transition from the muscle belly to the intersectio tendinea is gradually, and is located at the position of C4 for the cranial belly and at C8/C9 for the caudal belly. The cranial belly and the tendinous part have about the same length, but the caudal belly is clearly longer than the cranial belly. The sides of the cranial bellies of the M. biventer cervicis sinister and dexter touch each other dorsomedially. Most of the M. biventer cervicis is located dorsally to all other cervical muscles; only its origin and insertion are covered by other muscles. The connection schemes are simple, showing the extent of the muscles over the whole cervical column (Fig 2C and 2D).

*Origin*: This muscle originates from the most dorsal slip from the aponeurosis notarii, located dorsally of C14. The aponeurosis notarii is partly covered by thoracic muscles running laterocaudally (Fig 2A).

*Insertion*: The M. biventer cervicis inserts fleshy at the os supraoccipitale, with a broad but slender attachment site (Fig 2B). The insertion site is sunken at the os supraoccipitale located ventrally from the attachment site of the M. complexus (Fig 2B). The attachment sites of the M. complexus and M. biventer cervicis touch each other.

*Comparison*: Interestingly, this muscle is not present in all bird species (e.g., American darter (*Plotus anhinga*), flightless cormorant (*Phalacrocorax harrisi*, formerly *Nannopterum*), grey heron (*Ardea cinerea*) [7,11]), but was found in all four owls species examined by Boas (1929) [11]. In the M. biventer cervicis of the tawny owl (*Strix aluco*) the caudal belly is longer but narrower than the cranial belly, the latter is longer than the tendinous part [11].

*Specific discussion*: According to Kaupp (1918) the M. biventer cervicis raises the neck and extends the head relative to the trunk [31]. Since it runs along the whole S-shaped cervical column, a role in dorsal flexion of the head (summarised in [30]) is also suggested in *T*. *f*. *pratincola*. This muscle might thus help the owl to keep the head in an upright position as it uses it for parallax movements [26] or when it is alert and listening. The cranial belly covers the first and second osteological regions, the intersectio tendinea is located above the third and fourth osteological region, and the caudal belly covers the two most caudal osteological regions [5].

#### M. splenius capitis

**Fig 3:**
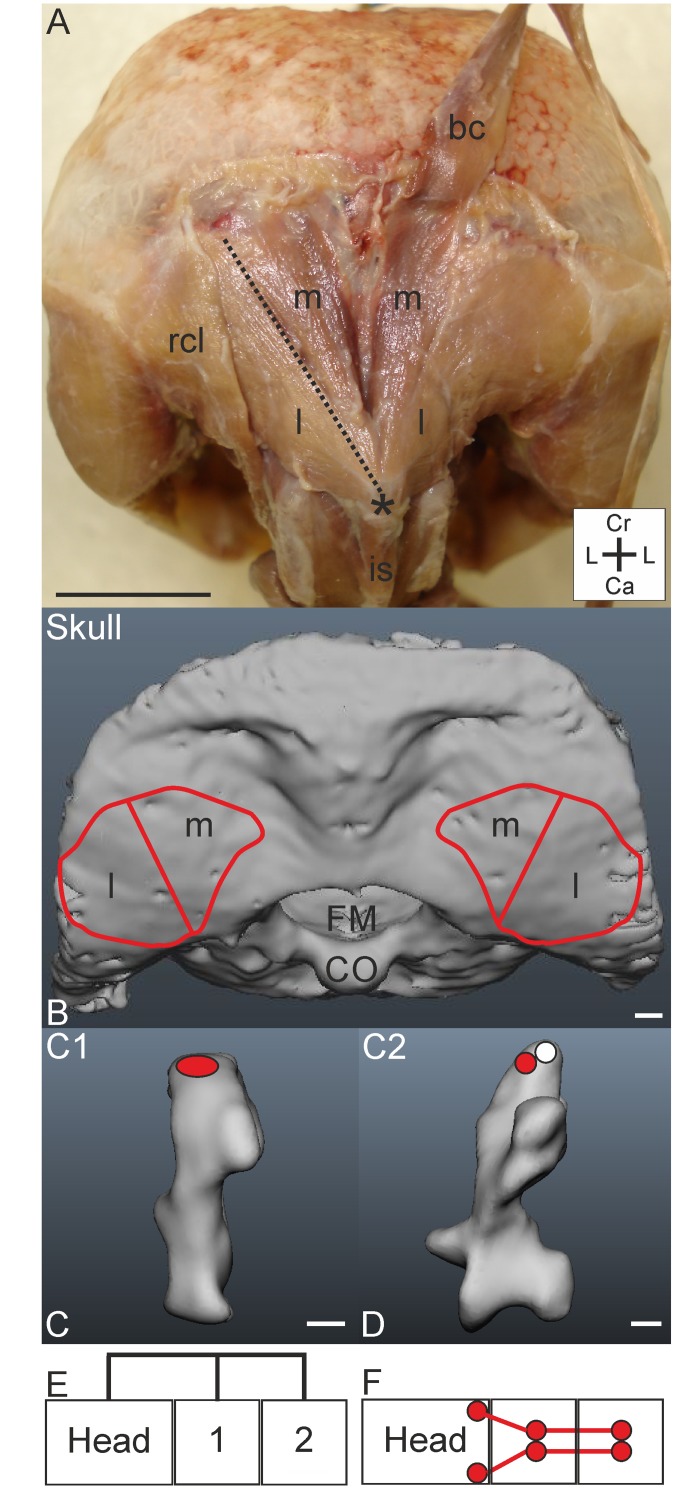
M. splenius capitis. A) Dorsal view on M. splenius capitis of *T*. *f*. *pratincola*. The origin from C2 is indicated by an asterisk. The left cranial belly of the M. biventer cervicis was removed to expose the M. splenius capitis, whereas the right cranial belly of the M. biventer cervicis (bc) was left intact and flapped back cranially. From the M. splenius capitis a pars medialis (m) and a pars lateralis (l) could be distinguished and were separated by a thin fascial sheath, which is indicated by a broken line in the M. splenius capitis sinister. The M. splenius capitis inserts ventrally to the M. biventer cervicis (bc) and medially to the M. rectus capitis lateralis (rcl). Coordinate system indicates cranial (Cr), lateral (L) and caudal (Ca). Scale bar represents one centimetre. B) Dorsal view on the skull with indicated insertion sites of the M. splenius capitis, pars medialis (m) and pars lateralis (l). Foramen magnum (FM) and condylus occipitalis (CO) are indicated. Scale bar represents one millimetre (adapted from [5]). C-D) Originating muscle attachment sites of the M. splenius capitis (coloured areas) in the three-dimensional models of the vertebrae of *T*. *f*. *pratincola*. In D the red area represents the origin of the pars medialis and the white area the origin of the pars lateralis. View on the vertebrae (C1-C2) from lateral left; cranial is left. Scale bars in C-D represent one millimetre (adapted from: [5]). E) Connection diagram from lateral view of M. splenius capitis in *T*. *f*. *pratincola*; origin and insertion sites are connected with lines representing the muscle slips. F) Connection diagram from dorsal view of M. splenius capitis in which the muscle attachment sites are indicated with red circles and are interconnected by a line representing the muscle slips.

*Muscle characteristics*: Within the M. splenius capitis in *T*. *f*. *pratincola* a pars lateralis and a pars medialis could be distinguished (Fig 3A). Both the pars medialis and pars lateralis originate from C2 (Fig 3A) and diverge when they run cranially before insertion on the os supraoccipitale (Fig 3A). The M. splenius capitis pars medialis is also connected with the dorsal side of the arcus vertebrae of C1 (Fig 3C, 3E and 3F). Both parts are positioned oblique in respect to the craniocaudal axis of the cervical column, and have parallel oriented fibres. The M. splenius capitis is approximately two centimetres in length, measured from the origin on C2 to the most cranial insertion site on the skull. The connection diagram from dorsal view shows the oblique course of this muscle (Fig 3F).

*Origin*: The pars medialis originates dorsocranially from the processus spinosus of C2 (red area Fig 3D) and dorsocranially from the arcus vertebrae of C1 (red area in Fig 3C). The pars lateralis originates dorsomedially from the processus spinosus of C2 (white area in Fig 3D) touching the origin of the pars medialis. Both parts originate fleshy (Fig 3A).

**Fig 4:**
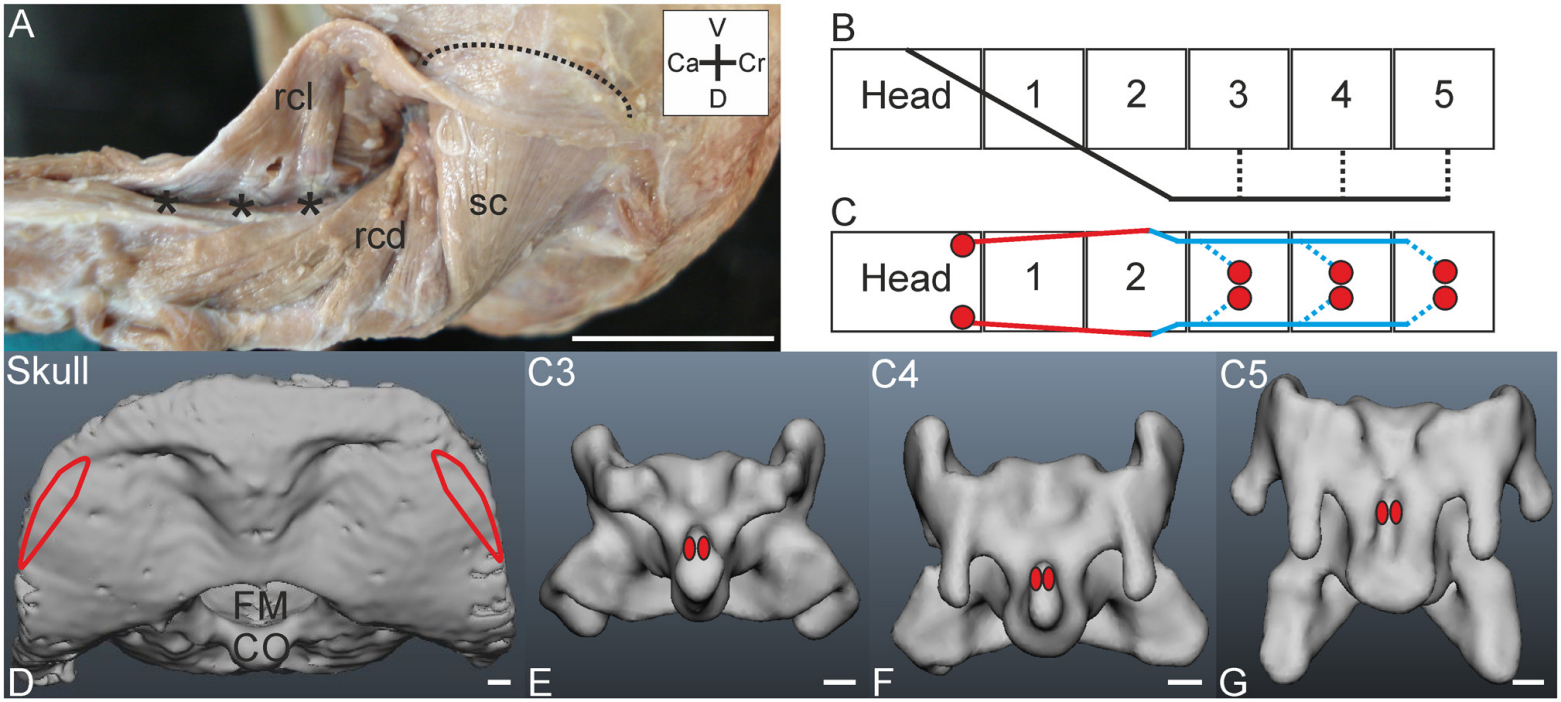
M. rectus capitis lateralis. A) Dorsolateral view on the M. rectus capitis lateralis (rcl). The muscle originates ventrally from C3-C5 (indicated by asterisks), wraps around the neck and inserts on the cranium. The M. rectus capitis lateralis is located superficially from the M. splenius capitis (sc) and M. rectus capitis dorsalis (rcd). Coordinate system indicates ventral (V), cranial (Cr), dorsal (D) and caudal (Ca). Scale bar represents one centimetre. B) Connection diagram from lateral view of M. rectus capitis lateralis in *T*. *f*. *pratincola*; origin and insertion sites are connected with lines representing the muscle slips, broken lines indicate tendinous parts. C) Connection diagram from dorsal view of M. rectus capitis lateralis in which the muscle attachment sites are indicated with red circles and are interconnected by a line representing the muscle slips. The blue lines represent ventrally located slips, which are thus actually behind the field of vision in a dorsal view. D) Dorsal view on the skull with indicated insertion sites of the M. rectus capitis lateralis. Foramen magnum (FM) and condylus occipitalis (CO) are indicated. Scale bar represents one millimetre (adapted from [5]). E-G) Origins of the M. rectus capitis lateralis indicated with red ellipses in the three-dimensional models of the vertebrae of *T*. *f*. *pratincola* from ventral view (cranial is on top) of vertebrae (C3-C5). Scale bars in E-G represent one millimetre (adapted from [5]).

*Insertion*: The pars medialis inserts ventrally from the insertion site of the M. biventer cervicis on the os supraoccipitale (Fig 3A). The insertion site of the pars lateralis is at the os supraoccipitale, lateromedially to that of the M. rectus capitis lateralis (Fig 3B). The insertion points touch the attachment site of the M. rectus capitis lateralis (Fig 4). The area of attachment of the M. splenius capitis on the cranium is huge compared with those of the M. complexus (Fig 1) and M. biventer cervicis (Fig 2).

*Comparison*: In some bird species, like the bald eagle (*Haliaeetus leucocephalus*), the ostrich (*Struthio camelus*) [25] and the buzzard (*Buteo buteo*) [10], the M. splenius capitis was also subdivided in a lateral and medial part, which is in line with our observations on *T*. *f*. *pratincola*. In the mallard (*Anas platyrhynchos*) even three parts (i.e., dorsomedial, lateral and ventrolateral part) could be distinguished [22], but a third part could not be identified in *T*. *f*. *pratincola*. In apodiform birds the M. splenius capitis has a cruciform morphology [33] which varies between individuals [9]. We did not observe such morphology in any of the specimen of *T*. *f*. *pratincola* although this adapted morphology may facilitate fast head rotations [34]. An attachment site at C1 is not general, but is also documented for the ruby topaz (*Chrysolampis mosquitus*) and the edible-nest swift (*Aerodramus fuciphagus*) [34] and the common swift (*Apus apus*) [9].

*Specific discussion*: A simultaneous contraction of the M. splenius capitis sinister and dexter moves the head dorsally, whereas unilateral contraction causes the head to move to a side [16]. A function of the M. splenius capitis in fast rotatory head movements was suggested [33,34]. This may also be the role in *T*. *f*. *pratincola*, because the origins on C1 and C2 are located more medially in relation to the insertion point on the cranium which reaches more lateral regions (Fig 3A), giving the short muscle a possibility to pull on the cranium and to induce rotations about the vertical axis. It may also underlie large head movements. The muscle originates from the most cranially located vertebrae which belong to the first and second osteological region [5].

#### M. rectus capitis lateralis

*Muscle characteristics*: In *T*. *f*. *pratincola* the parallel fibred M. rectus capitis lateralis (Fig 4) originates ventrally from C3, C4 and C5 (Fig 4E–4G). The slips fuse ventrolaterally and travel cranially along the ipsilateral side until they end dorsally and insert on the cranium (Fig 4A and 4D). This muscle is approximately three centimetres in length, measured from the most caudal point of origin. The origins on the cervical vertebrae are all ventromedial (Fig 4E–4G), while the insertion site at the cranium is dorsolateral (Fig 4A–4D) and elongated dorsoventrally.

*Origin*: The M. rectus capitis lateralis originates from muscle slips attaching with a tendon to the processus ventralis of C3, C4 and C5 (Fig 4E–4G). The originating slips of the M. rectus capitis lateralis come together with the slips of the M. rectus capitis ventralis and attach tendinously at the processus ventralis of vertebrae C3, C4 and C5.

*Insertion*: This muscle inserts fleshy on the cranium (Fig 4A), laterally from the attachment site of the M. splenius capitis (Fig 4D). The dorsal tip of the attachment site of the M. rectus capitis lateralis touches the lateral tip of the attachment site of the M. complexus which is located more dorsomedially. Though the insertion sites of the M. complexus and M. rectus capitis lateralis touch each other, the muscles are clearly not fused and have individual insertions.

*Comparison*: In the mallard (*Anas platyrhynchos*) an origin from C2, C3 and C4 was observed [22]. An attachment site on C2 was also documented in the common buzzard (*Buteo buteo*) [10], but an origin from C2 could not be confirmed in *T*. *f*. *pratincola*. However, the origin of this muscle from C3, C4 and C5 in *T*. *f*. *pratincola* corresponds with the arrangement in the pigeon (*Columba livia*) [14] and the raven (*Corvus corax sinuatus*) [13]. This muscle may be responsible for head movements to the side and flexion of the neck [14].

*Specific discussion*: The vertebrae which give rise to this muscle belong to the second and third osteological region in *T*. *f*. *pratincola* [5]. The M. rectus capitis lateralis was suggested to be involved in lateral flexion of the head [25]. This may also be the function in *T*. *f*. *pratincola*, and the muscle may underlie the large rotational capability of barn owls that can roll their heads easily up to 90 degrees (see Fig 17 in [35]).

#### M. rectus capitis ventralis

**Fig 5:**
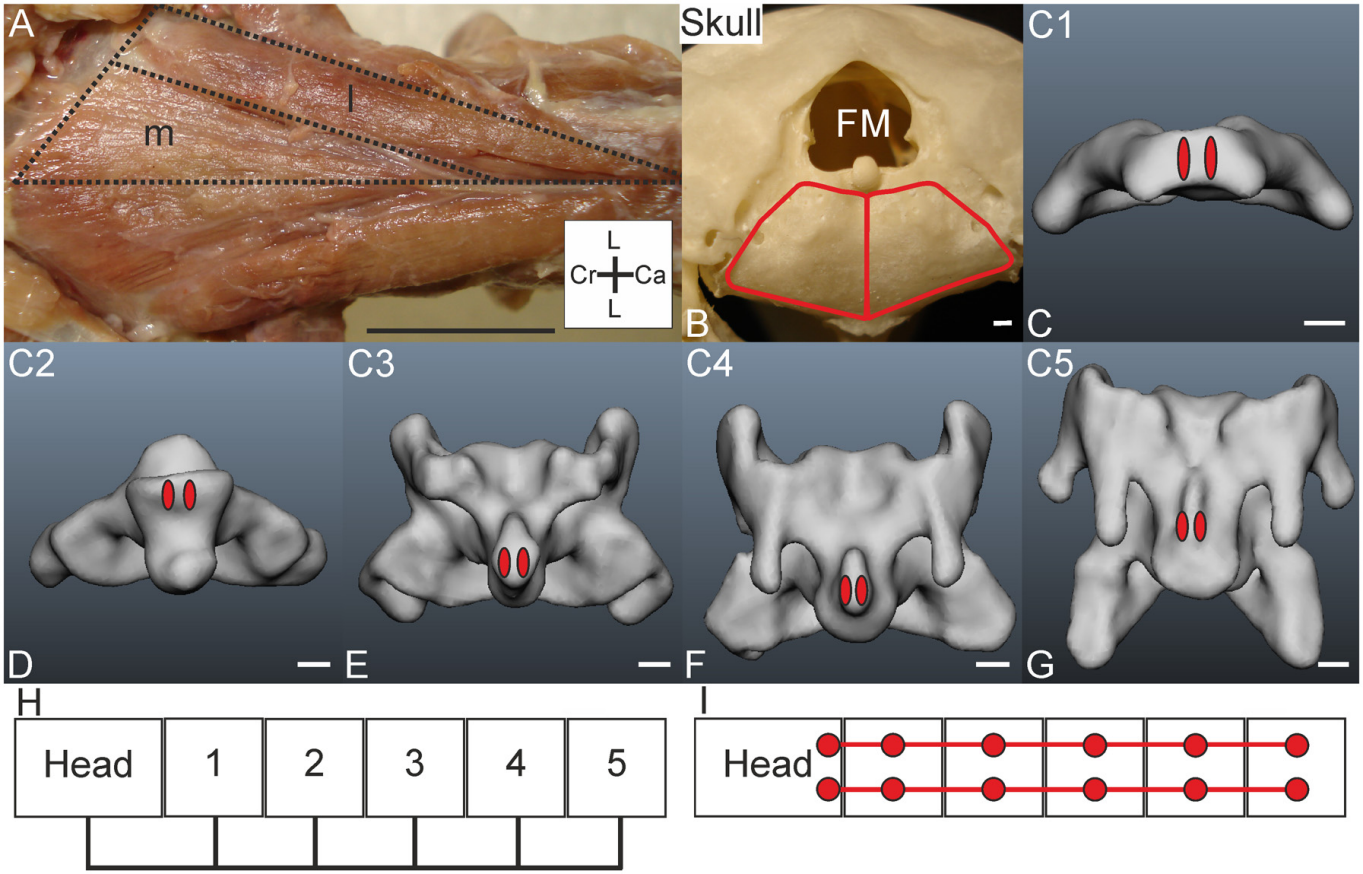
M. rectus capitis ventralis. A) Craniocervical region from ventral view showing the M. rectus capitis ventralis from which the M. rectus capitis ventralis sinister, pars lateralis (l) and pars medialis (m) are bordered by a broken line. Coordinate system indicates lateral (L), caudal (Ca) and cranial (Cr). Scale bar represents one centimetre. B) Skull from caudal view with indicated foramen magnum (FM) and insertion site of M. rectus capitis ventralis on the os basioccipitale (red line). Scale bar represents one millimetre. C-G) Muscle attachment sites of the M. rectus capitis ventralis indicated with red ellipses in the three-dimensional models of the vertebrae of *T*. *f*. *pratincola*: ventral view (cranial is on top). Scale bars in C-G represent one millimetre (adapted from [5]). H) Connection diagram from lateral view of M. rectus capitis ventralis in *T*. *f*. *pratincola*; origin and insertion sites are connected with lines representing the muscle slips. I) Connection diagram from dorsal view of M. rectus capitis ventralis in which the muscle attachment sites are indicated with red circles and are interconnected by a line representing the muscle slips.

*Muscle characteristics*: In *T*. *f*. *pratincola* the M. rectus capitis ventralis (Fig 5) originates ventrally from C1-C5 (Fig 5C–5G) and inserts on the os basioccipitale (Fig 5B). A pars lateralis and a pars medialis could be distinguished (Fig 5A). The parts are separated by the internal carotid arteries which run cranially. In *T*. *f*. *pratincola*, the two parts were strongly interconnected (Fig 5A). The length of the muscle fibres varies along the muscle, but the longest fibres (which belong to the pars lateralis), span a length of approximately three centimetres.

*Origin*: This muscle originates from the processus ventralis of C1-C5 (Fig 5C–5G). The pars medialis originates from cranially located cervical vertebrae. Although the origin at C1 and C2 could be clearly identified, and there is also an attachment site at C3, it remained unclear whether this attachment site belonged to the pars medialis or to the pars lateralis. The pars lateralis originates from the more caudally located vertebrae, C4 and C5. As mentioned before, it may also have an origin at C3.

*Insertion*: The insertion points of the pars medialis on the skull are at the os basioccipitale. The pars lateralis inserts more laterally at the os basioccipitale than the pars medialis. The insertion sites of both parts are fleshy.

*Comparison*: In the mallard (*Anas platyrhynchos*) the pars medialis originates from C1 and C2, and the pars lateralis originates from C3-C6 [22]. In the whooping crane (*Grus americana*) an asymmetrical arrangement was documented: the M. rectus capitis ventralis, pars lateralis sinister attaches at C5, whereas the pars lateralis dexter only reaches C4 [15]. In the common buzzard (*Buteo buteo*) the origin of this muscle remained partly unclear, because the neck was not completely preserved [10]. However, in this species also, the pars lateralis and pars medialis fuse at the level of C3 [10].

*Specific discussion*: Cervical vertebrae of osteological regions 1–3 [5] serve as muscle attachment site of this muscle. Due to its arrangement, this muscle may underlie ventroflexion (forward pitching) of the head [32]. The muscle has a function in ventro- and lateroflexion (see summary in [30]). Compared to the M. longus colli ventralis, the function in ventroflexion may be limited, but may nevertheless highly contribute to head rotations in owls. Snively et al. (2014) [32] suggest that in *T*. *f*. *pratincola* this muscle may have a role in the fixation phase during hunting [26].

#### M. rectus capitis dorsalis

**Fig 6:**
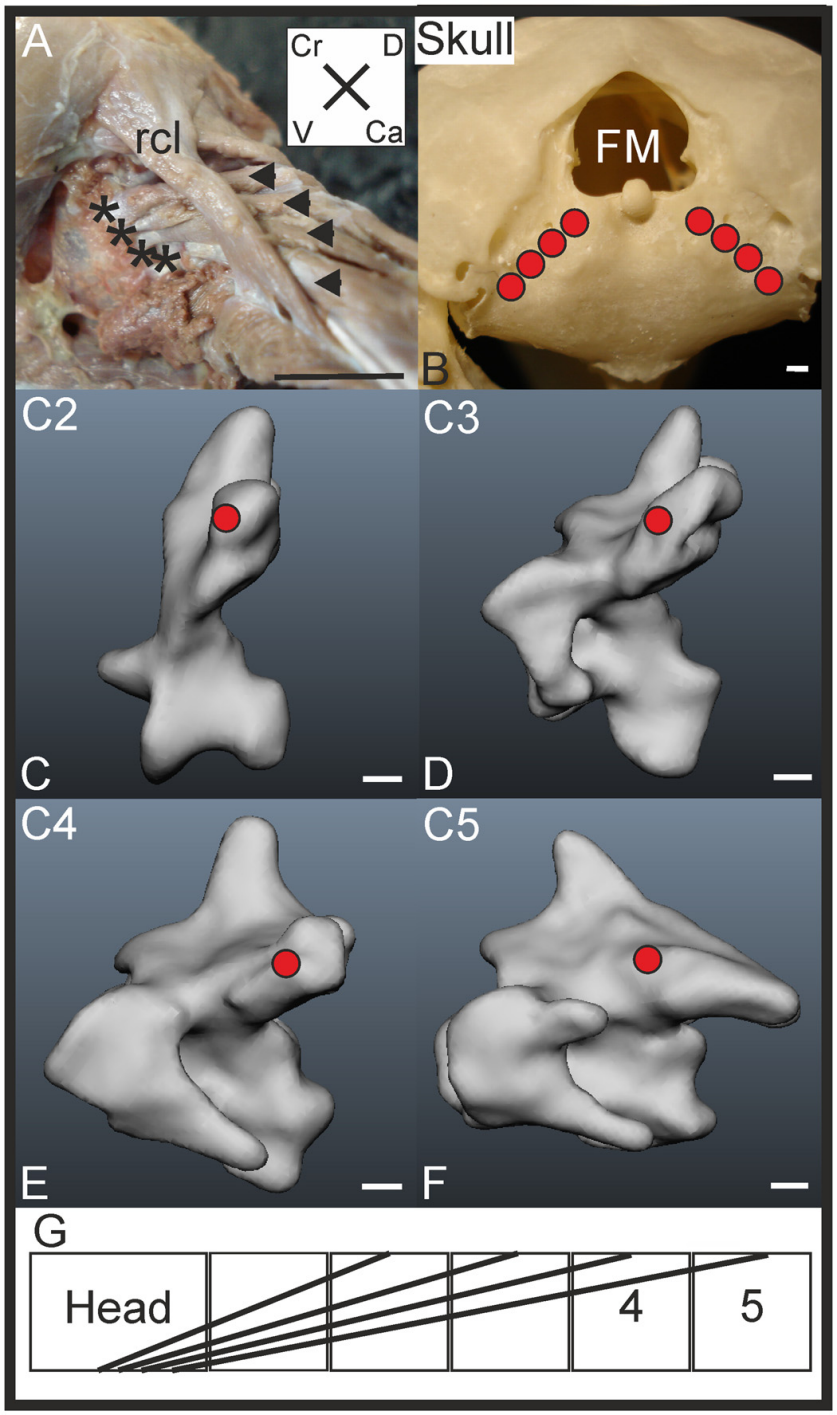
M. rectus capitis dorsalis. A) Lateral view on M. rectus capitis dorsalis. The insertion points on the cranium are indicated by asterisks. Note the parallel orientation of the four muscles (indicated with arrowheads). The slips of the M. rectus capitis dorsalis are located deeply from the M. rectus capitis lateralis (rcl). Coordinate system indicates dorsal (D), caudal (Ca), ventral (V) and cranial (Cr). Scale bar represents one centimetre. B) Skull from caudal view with indicated foramen magnum (FM), and insertion sites of M. rectus capitis dorsalis indicated with red filled circles. C-F) Origin sites of M. rectus capitis dorsalis indicated with red circles in the three-dimensional models of the vertebrae of *T*. *f*. *pratincola*: lateral left view (cranial is left). Scale bars in C-F represent one millimetre (adapted from [5]). G) Connection diagram from lateral view of *T*. *f*. *pratincola*; origin and insertion sites are connected with lines representing the muscle slips.

*Muscle characteristics*: This muscle was comprised of four individual slips (Fig 6A). The origins were dorsolaterally on the lateral processes of C2-C5 (Fig 6C–6F). The four slips run longitudinally in parallel along the ipsilateral side of the neck (Fig 6A). The slip originating at C2 takes the most cranial path (Fig 6A). The slip originating from C4 inserts dorsomedially to those of the other three slips, located just below the lateral edge of the foramen magnum. All slips are comprised of parallel organised muscle fibres.

*Origin*: The fleshy origin on the lateral processus of C2 (Fig 6C) is located dorsocranially whereas the M. longus colli dorsalis, pars caudalis inserts dorsocaudally at this processus. The other slips originate fleshy from the lateral processus of C3-C5 (Fig 6D–6F).

*Insertion*: The attachment sites to the os basioccipitale are tendinous and completely covered by the M. rectus capitis ventralis. The slips of the M. rectus capitis dorsalis are *in situ* covered by the M. rectus lateralis. The slip originating from C2 inserts most laterally whereas each following slip (originating from the next vertebra) inserts more medially and caudally than the previous one. The dorsoventral course of the muscle can be clearly seen in the connection diagram from lateral view (Fig 6G).

*Comparison*: In the mallard (*Anas platyrhynchos*) this muscle has five slips originating from C1-C5 [22]. An origin from C1 is also documented for the common buzzard (*Buteo buteo*) [10], but could not be identified in *T*. *f*. *pratincola*.

*Specific discussion*: The origins of this muscle fits well to the second osteological region extending from C2 to C4, C5 is located in the third region [5]. The arrangement of this muscle suggests an antagonistic function to the M. rectus capitis lateralis.

### Cervical muscles region 2: Mm. cervicales dorsales

#### M. longus colli dorsalis

**Fig 7:**
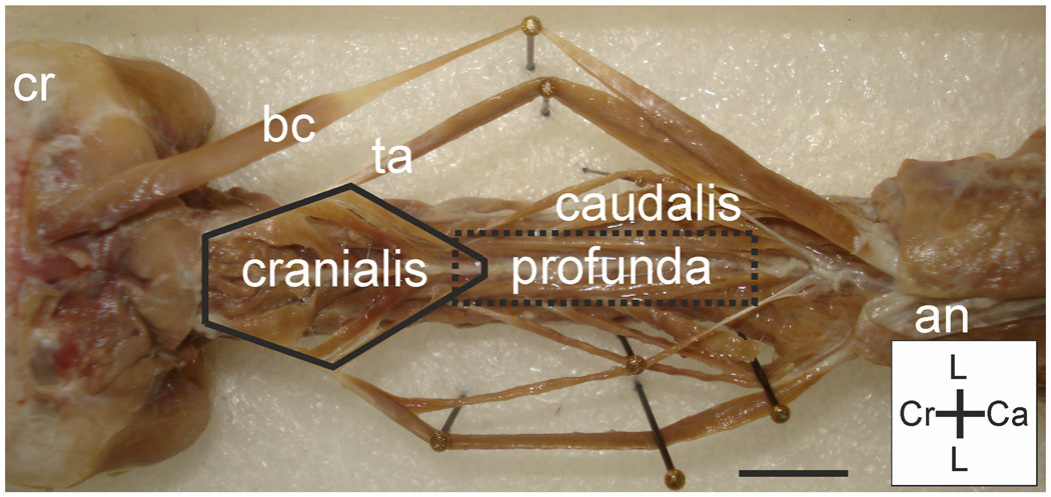
M. longus colli dorsalis overview. Dorsal view on M. longus colli dorsalis: pars cranialis (bordered by solid line) is located cranially and connected to the more caudally located pars caudalis by the tendo axialis (ta). The pars profunda (surrounded by dotted rectangle) is located ventrally from the pars caudalis. All parts are located ventrally from the M. biventer cervicis (bc). Cranium (cr) and aponeurosis notarii (an) are indicated for clarity. Coordinate system indicates lateral (L), caudal (Ca) and cranial (Cr). Scale bar represents one centimetre.

Baumel et al. (1993) [6] subdivided the M. longus colli dorsalis into four parts: pars cranialis, pars caudalis, pars profunda and pars thoracica [6]. However, descriptions of the M. longus colli dorsalis, pars thoracica has not yet been clarified and this muscle may belong to the M. longissimus dorsi [6]. Because the pars thoracica’s affinities are uncertain we will leave this part out of account and treat the M. longus colli dorsalis as one muscle that has three different parts (i.e., pars cranialis, pars caudalis and pars profunda) (Fig 7), following Landolt & Zweers (1985) [22]. For clarity the individual parts are discussed separately. In *T*. *f*. *pratincola* the M. longus colli dorsalis (*sensu lato*) is a complex muscle (Fig 7) with many slips and interconnections and takes a considerable volume of the Mm. cervicales dorsales (muscle region 2).

#### M. longus colli dorsalis, pars caudalis

**Fig 8:**
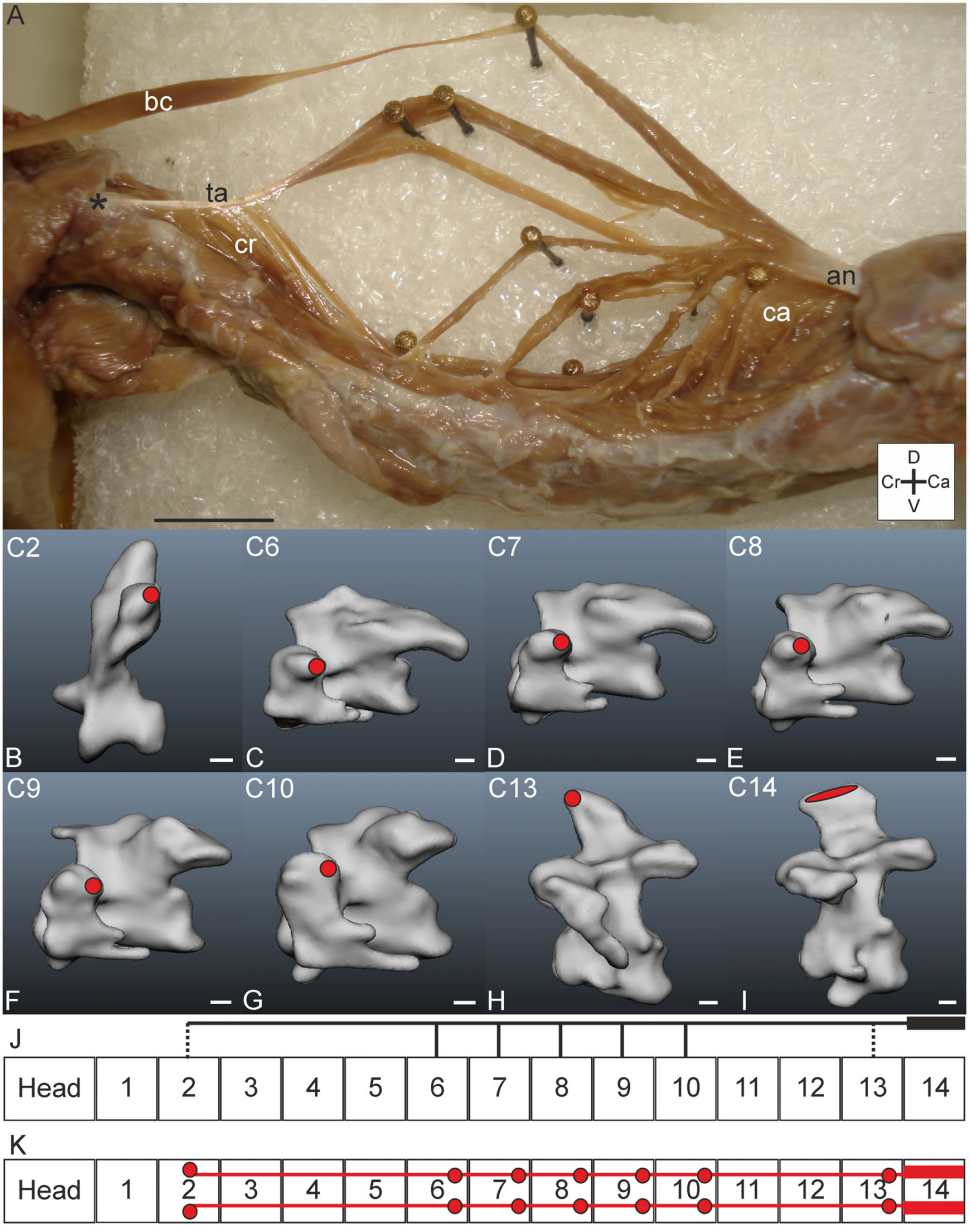
M. longus colli dorsalis, pars caudalis. A) Lateral view. M. longus colli dorsalis, pars caudalis is located ventrally from the M. biventer cervicis (bc). The pars caudalis (ca) originates from the aponeurosis notarii (an) with one slip running cranially. It becomes tendinously (tendo axialis, ta) before insertion to C2 (indicated by an asterisk). The slips of the pars cranialis (cr) insert to the tendo axialis (ta). Coordinate system indicates dorsal (D), caudal (Ca), ventral (V) and cranial (Cr). Scale bar represents one centimetre. B-I) Muscle attachment sites of the M. longus colli dorsalis, pars caudalis indicated with red areas in the three-dimensional models of the vertebrae of *T*. *f*. *pratincola*: lateral view. Cranial is to the left. Scale bars in B-I represent one millimetre (adapted from: [5]). J) Connection diagram from lateral view of M. longus colli dorsalis, pars caudalis in *T*. *f*. *pratincola*; origin and insertion sites are connected with lines representing the muscle slips and broken line represents tendinous parts, heavy line represents aponeurosis notarii. K) Connection diagram from dorsal view of M. longus colli dorsalis, pars caudalis in which the muscle attachment sites are indicated with red circles and are interconnected by a line representing the muscle slips. The heavy lines represent the aponeurosis notarii.

*Muscle characteristics*: The pars caudalis (Fig 8) takes the largest volume of the M. longus colli dorsalis. Several slips originate unipennate from the aponeuris notarii and insert both at caudally located vertebrae and to more cranially located vertebrae; insertion sites reach as far as C2 (Fig 8A). All muscle fibres are organised in parallel. In the connection diagrams (Fig 8J and 8K) it can be seen that the slips of the pars caudalis cover a large part of the cervical column.

**Fig 9:**
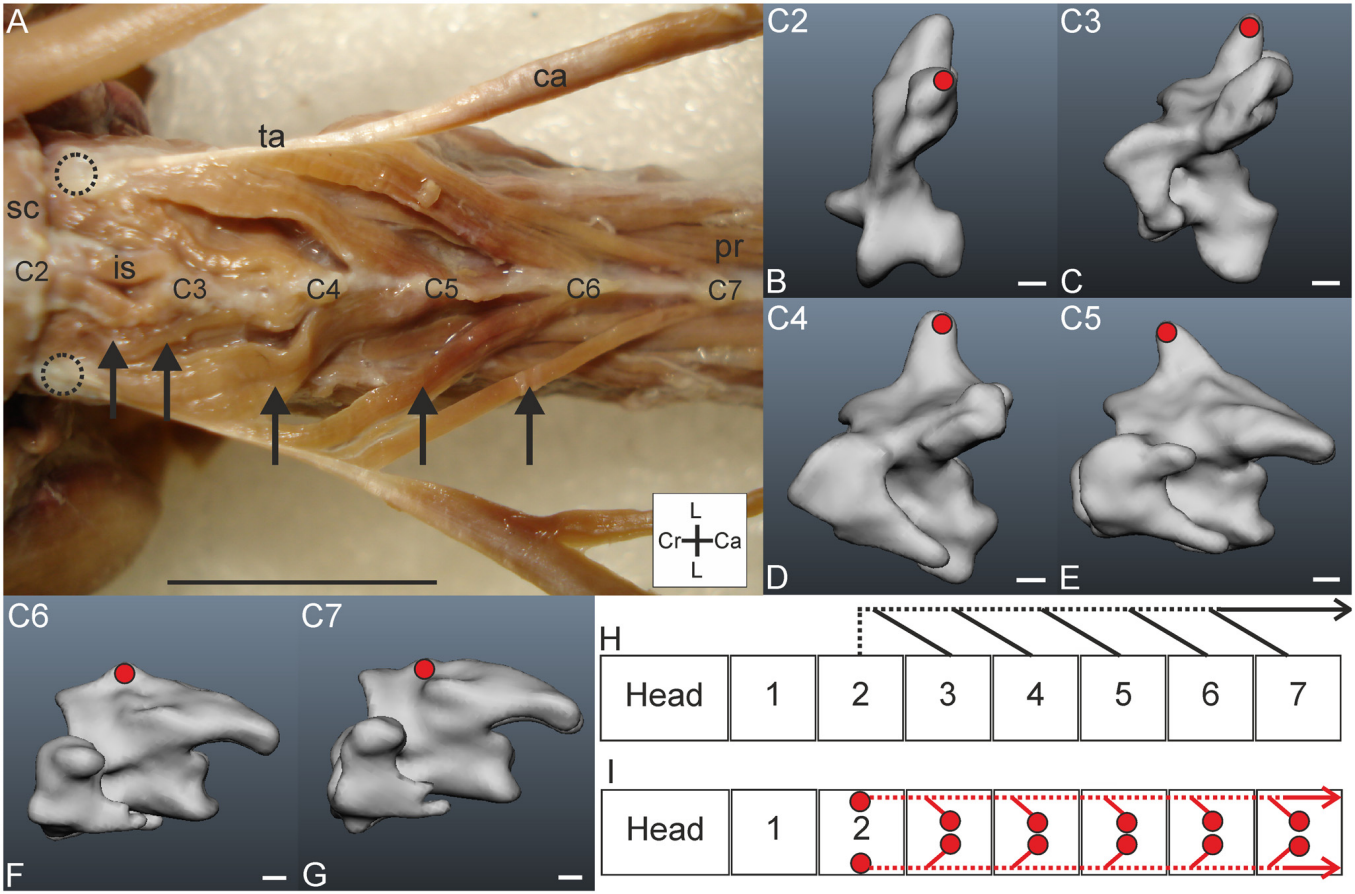
M. longus colli dorsalis, pars cranialis. A) Dorsal view on M. longus colli dorsalis, pars cranialis. Vertebrae numbers are indicated (C2-C7). The slips from the pars cranialis insert to the tendo axialis (ta) from a pars caudalis (ca) slip. The lateral processus of C2, which serve as insertion point for the tendo axialis are indicated with broken lines. The M. interspinalis (is), M. splenius capitis (sc) and M. longus colli dorsalis, pars profunda (pr) are indicated for reference. Coordinate system indicates lateral (L), caudal (Ca) and cranial (Cr). Scale bar represents one centimetre. B-G) Muscle attachment sites of the M. longus colli dorsalis, pars cranialis indicated with red circles in the three-dimensional models of the vertebrae of *T*. *f*. *pratincola* from dorsal view (cranial is on top). Scale bars in B-G represent one millimetre (adapted from [5]). H) Connection diagram from lateral view of M. longus colli dorsalis, pars cranialis in *T*. *f*. *pratincola*; origin and insertion sites are connected with lines representing the muscle slips, broken lines represent the tendo axialis. The arrowhead indicates that this muscle slip (from the M. longus colli dorsalis, pars caudalis) runs further caudally. I) Connection diagram from dorsal view of M. rectus capitis ventralis in which the muscle attachment sites are indicated with red circles and are interconnected by a line representing the muscle slips. Broken lines indicate the tendo axialis. The arrowheads indicate that this muscle slip (from the M. longus colli dorsalis, pars caudalis) runs further caudally.

*Origin and insertion*: The M. longus colli dorsalis, pars caudalis of *T*. *f*. *pratincola*, originates from the aponeurosis notarii (Fig 8A). This aponeurosis is located ventrally from the originating aponeurosis of the M. biventer cervicis. The aponeurosis notarii probably attaches to C14 (Fig 8I). A solid muscle belly originates from the aponeurosis notarii; this muscle belly runs cranially and inserts with a tendon (called tendo axialis) to the torus dorsalis (a lateral processus also known as epipophysis [25]) of C2 (Figs 8B and 9A). This muscle slip is approximately seven centimetres in length. From the processus spinosus of C13 (Fig 8H) a muscle slip originates tendinously and runs four centimetres cranially. This slip fuses with the tendo axialis which inserts on C2. Several other slips originate from the aponeurosis notarii and insert laterally on C6-C10 (Fig 8C–8G) and on more caudally located vertebrae with an interwoven network of inseparable muscle slips from which the insertion sites could not be identified precisely.

*Comparison*: The arrangement of the pars caudalis fits well to the description as described in Baumel et al. (1993) [6]. Baumel et al. (1993) mention that the M. longus colli dorsalis, pars caudalis, has several fleshy muscle slips originating from the aponeurosis notarii and processus spinosi of the last cervical vertebrae [6]. They also state that the lateral slips shade off into tendons with insertions on the torus dorsalis of the second and third region as defined by Boas (1929) [6].

*Specific discussion*: In osteology, the cervical vertebrae involved in the M. longus colli dorsalis, pars caudalis belong to different regions; all regions are covered except for the first region (consisting of C1) [5].

#### M. longus colli dorsalis, pars cranialis

*Muscle characteristics*: In *T*. *f*. *pratincola* the M. longus colli dorsalis, pars cranialis (Fig 9) was identified as five parallel fibred slips originating from C3-C7 (Fig 9A and 9C–9G), which insert unipennate to the tendo axialis (Fig 9A). The slips originating from more caudal vertebrae are longer than the slips originating from the more cranial vertebrae. The connection diagrams (Fig 9H and 9I) show the dorsal path and the unipennate insertion to the tendo axialis.

*Origin*: Five slips originate fleshy from the processus spinosi of C3-C7 and run cranially (Fig 9C–9G).

*Insertion*: The slips insert at the tendo axialis from the M. longus colli dorsalis, pars caudalis which is attached to the torus dorsalis of C2 (Fig 9A and 9B). Compared with the origin of the M. rectus capitis dorsalis (Fig 6C), the insertion point of the M. longus colli dorsalis, pars caudalis is more caudally on the processus transversus of C2.

*Comparison*: Boas (1929) described this muscle as M. splenius colli and documented six slips in the tawny owl (*Strix aluco*), of which the first slip was strong and the second and sixth were weak [11]. A similar arrangement as found in *T*. *f*. *pratincola* was also documented for more distantly related species, e.g., noisy scrubbird (*Atrichornis clamosus*) and the superb lyrebird (*Menura novaehollandiae*) [21].

*Specific discussion*: This muscle’s dorsal location likely involves it in head protraction, as was found also in electromyography studies in the chicken (*Gallus gallus domesticus*) [32]. This muscle also forms a chain with the M. rectus capitis dorsalis, interacting closely at C2. The slips cover the second and third osteological regions [5].

#### M. longus colli dorsalis, pars profunda

**Fig 10:**
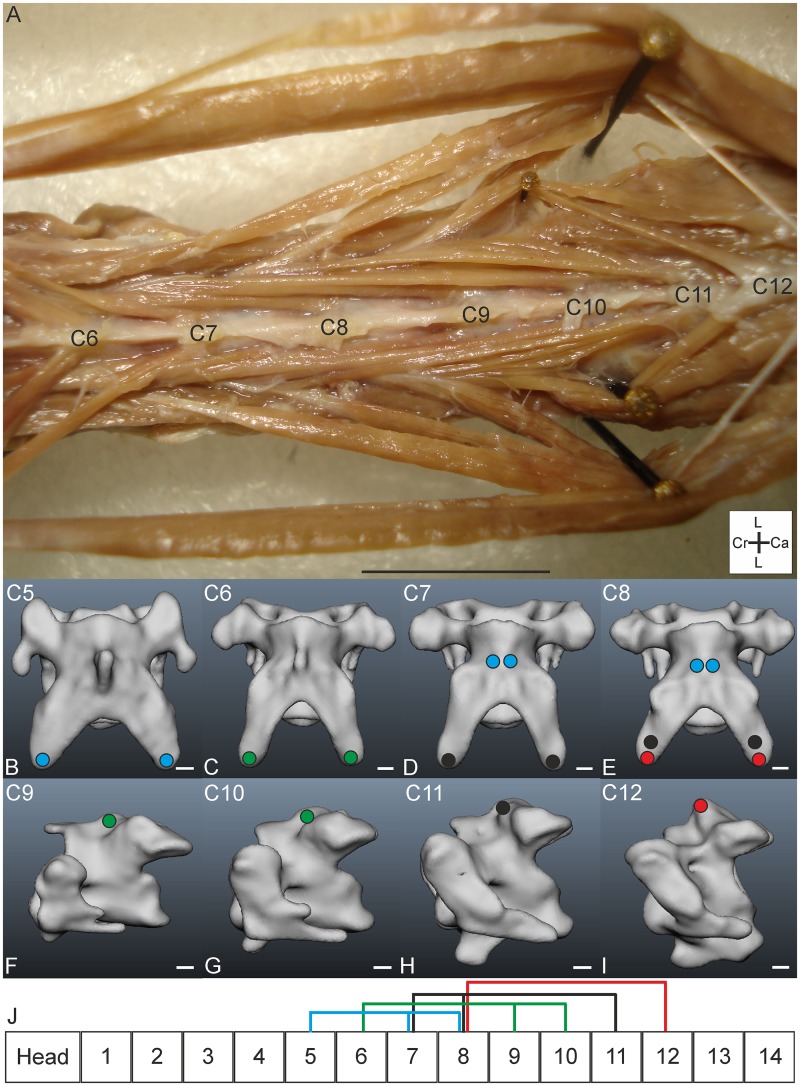
M. longus colli dorsalis, pars profunda. A) Dorsal view on M. longus colli dorsalis, pars profunda. Due to its deep location more dorsally located muscle slips were spread apart by needles. The numbers of the cervical vertebrae are indicated (C6-C12). Coordinate system indicates lateral (L), caudal (Ca) and cranial (Cr). Scale bar represents one centimetre. B-I) Attachment sites of the individual M. longus colli dorsalis, pars profunda slips. Vertebrae in B-E are shown in dorsal view in which cranial is on top, vertebrae in F-I are shown in lateral view in which left is cranial. The colours of the circles indicate to which slip the attachment site belongs and corresponds with the colours as used in the connection diagram (J). Scale bars in B-I represent one millimetre (adapted from: [5]). J) Connection diagram from lateral view of M. longus colli dorsalis, pars profunda in *T*. *f*. *pratincola*; origin and insertion sites are connected with lines representing the muscle slips. Colours are given for clarity and represent slips from the same muscle and correspond to the colours in B-I.

*Muscle characteristics*: The M. longus colli dorsalis, pars profunda of *T*. *f*. *pratincola* (Fig 10) consists of several individual muscles which are located within C5-C12 (Fig 10B–10I). The pars profunda is located ventrally from the pars caudalis. The delicate muscles pass one or more vertebrae before they insert (Fig 10A). The muscles have parallel fibres and the individual muscles are also oriented parallel from each other.

*Origin and insertion*: The individual slips originate from the processus spinosus of C7-C12, respectively. Insertions are found on the processus transversus of C5-C8. The most caudal muscle (Fig 10J, red line) originates from the lateral side of the processus spinosus of C12 (Fig 10I) and inserts on C8 (Fig 10E (red area)). The muscle (Fig 10J, black line) that originates from C11 (Fig 10H) splits and inserts on C7 and C8 (Fig 10D and 10E (black area)). The slips (Fig 10J, green line) originating from C9 and C10 (Fig 10F and 10G) run cranially and fuse before they insert on C6 (Fig 10C). The same holds true for the slips (Fig 10J, blue line) originating from C7 and C8 (Fig 10D and 10E (blue area)) which fuse before they insert at C5 (Fig 10B).

*Comparison*: The M. longus colli dorsalis, pars profunda was described by Boas (1929) as Mm. dorsales pygmaei, and this author reported that this muscle was more developed in owls he dissected (i.e., tawny owl (*Strix aluco*), snowy owl (*Bubo scandiacus*), eagle owl (*Bubo bubo*), short-eared owl (*Asio flammeus*)) than in any other bird species he examined [11]. In the tawny owl (*Strix aluco*) this muscle originates from the processus spinosus of C7 to C13 and has insertion points from C4 to C11 [11]. The slip originating from C9 splits and inserts on C5 and C6, respectively. Boas (1929) also mentions a close connection between the muscles [11], which was also true for *T*. *f*. *pratincola*. The slips of the pars profunda in the huia (*Heteralocha acutirostris*), which has also fourteen cervical vertebrae, are restricted to more caudally vertebrae and reach from C8-C12 [27]. In the mallard (*Anas platyrhynchos*) the muscles of the pars profunda span two or three vertebrae [22], and in the chicken (*Gallus gallus*) one vertebra is skipped between origin and insertion [2]. The pars profunda is not present in all bird species and is lacking, for example, in both the great spotted woodpecker (*Dendrocopos major*) and middle spotted woodpecker (*Dendrocopos medius*) [19]. A function in upward flexion of vertebrae of the middle region of the neck was suggested earlier [16].

*Specific discussion*: In *T*. *f*. *pratincola* this muscle has origins and insertion points at C5-C12, a group of cervical vertebrae from the middle of the cervical column that form the third, fourth and fifth osteological region as determined by cluster analysis [5]. The fact that these muscles are absent in distantly related species (*Dendrocopos* spp.) [19], but are strongly developed in Strigiformes [11] may hint to a function in specific behaviour.

#### M. interspinalis

**Fig 11:**
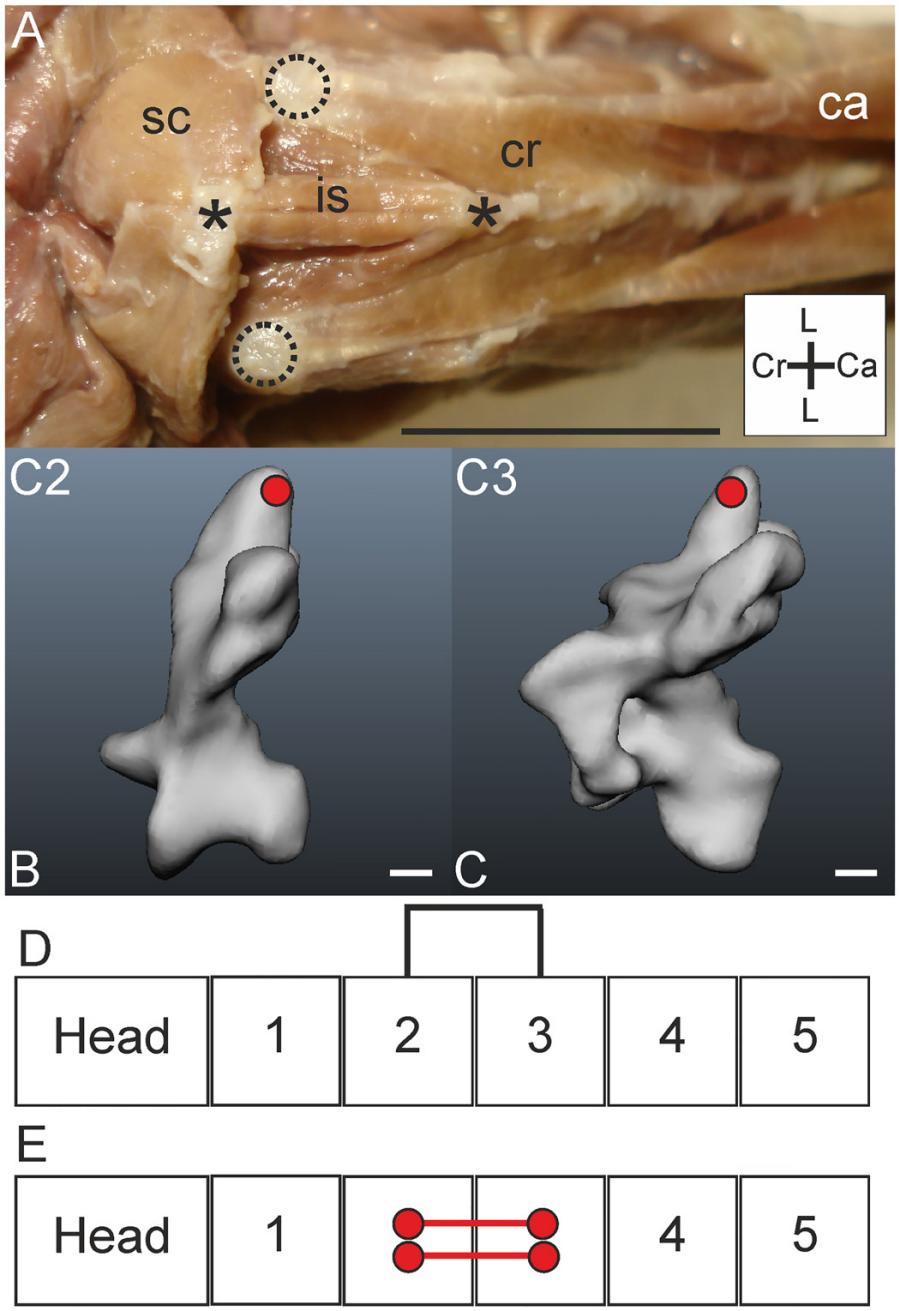
M. interspinalis. A) Dorsal view on M. interspinalis (is) spanning the space between the processus spinosus of C2 (insertion) and C3 (origin) which are indicated by asterisks whereas the tori dorsales of C2 are indicated by circles with broken lines. The M. splenius capitis (sc), M. longus colli dorsalis, pars cranialis (cr) and a slip from the M. longus colli dorsalis, pars caudalis (ca) are indicated as a reference. Coordinate system indicates lateral (L), caudal (Ca) and cranial (Cr). Scale bar represents one centimetre. B-C) Muscle attachment sites of the M. interspinalis indicated with red circles in the three-dimensional models of the vertebrae of *T*. *f*. *pratincola*: lateral view (cranial is to the left). Scale bars represent one millimetre (adapted from [5]). D) Connection diagram from lateral view of the M. interspinalis origin and insertion sites are connected with a line representing the muscle. E) Connection diagram from dorsal view of M. interspinalis in which the muscle attachment sites are indicated with red circles and are interconnected by a line representing the muscle.

*Muscle characteristics*: The Mm. interspinales consist of small dorsomedial slips, which connect the processus spinosi of adjacent vertebrae [6]. In *T*. *f*. *pratincola* a M. interspinalis (Fig 11) was identified as a short muscle located between the processus spinosi of C2 and C3 (Fig 11A). We did not further check whether there exist M. interspinales between other cervical vertebrae. The M. interspinalis is located medioventrally from the M. longus colli dorsalis, pars cranialis slips. The fibres of this muscle are oriented parallel and are approximately seven millimetres in length. Both origin and insertion are fleshy. The course of the muscle is simple and very short (Fig 11A, 11D and 11E).

*Origin*: This muscle originates medially from the processus spinosus of C3 (Fig 11C).

*Insertion*: The muscle inserts caudally at the processus spinosus of C2 (Fig 11B). This insertion site on the processus spinosus of C2 is caudomedial from the originating attachment site of the M. splenius capitis (Fig 11A).

*Comparison*: The occurrence of this muscle in *T*. *f*. *pratincola* is in line with the findings in the tawny owl (*Strix aluco*) [11] and the common buzzard (*Buteo buteo*) [10]. In the huia (*Heteralocha acutirostris*) three Mm. interspinales were observed located between C2-C5 [27].

*Specific discussion*: Contraction of these muscles cause adjacent processus spinosi to come closer [31], thus this muscle is involved in dorsiflexion. Because of its size the effect of a single muscle may be limited, but a simultaneous action with other muscles—also with other interspinalis muscles, if present—may result in a considerable dorsiflexion of a series of adjacent vertebrae. Unilateral contraction may lead to lateroflexion of the involved vertebrae to the side of contraction. The M. interspinalis in *T*. *f*. *pratincola*, connecting C2 and C3, is located in the second osteological region [5].

### Cervical muscles region 3: Mm. cervicales laterales

This laterally located subsystem is fused with the Mm. iliocostalis et longissimus dorsi [6]. The Mm. intertransversarii, Mm. inclusi and M. flexor colli lateralis and M. flexor colli medialis belong to this lateral subsystem [6]. In *T*. *f*. *pratincola* a thick and interconnected fascia in the lateral region hinders dissection and makes it hard to isolate individual muscles without affecting them. Furthermore, these muscles have a small appearance and a deep and difficult location. Therefore, none of these muscles were selected for examination in this study. For more information we refer the reader to Baumel et al. (1993) [6].

### Cervical muscles region 4: Mm. cervicales ventrales

#### M. longus colli ventralis

**Fig 12:**
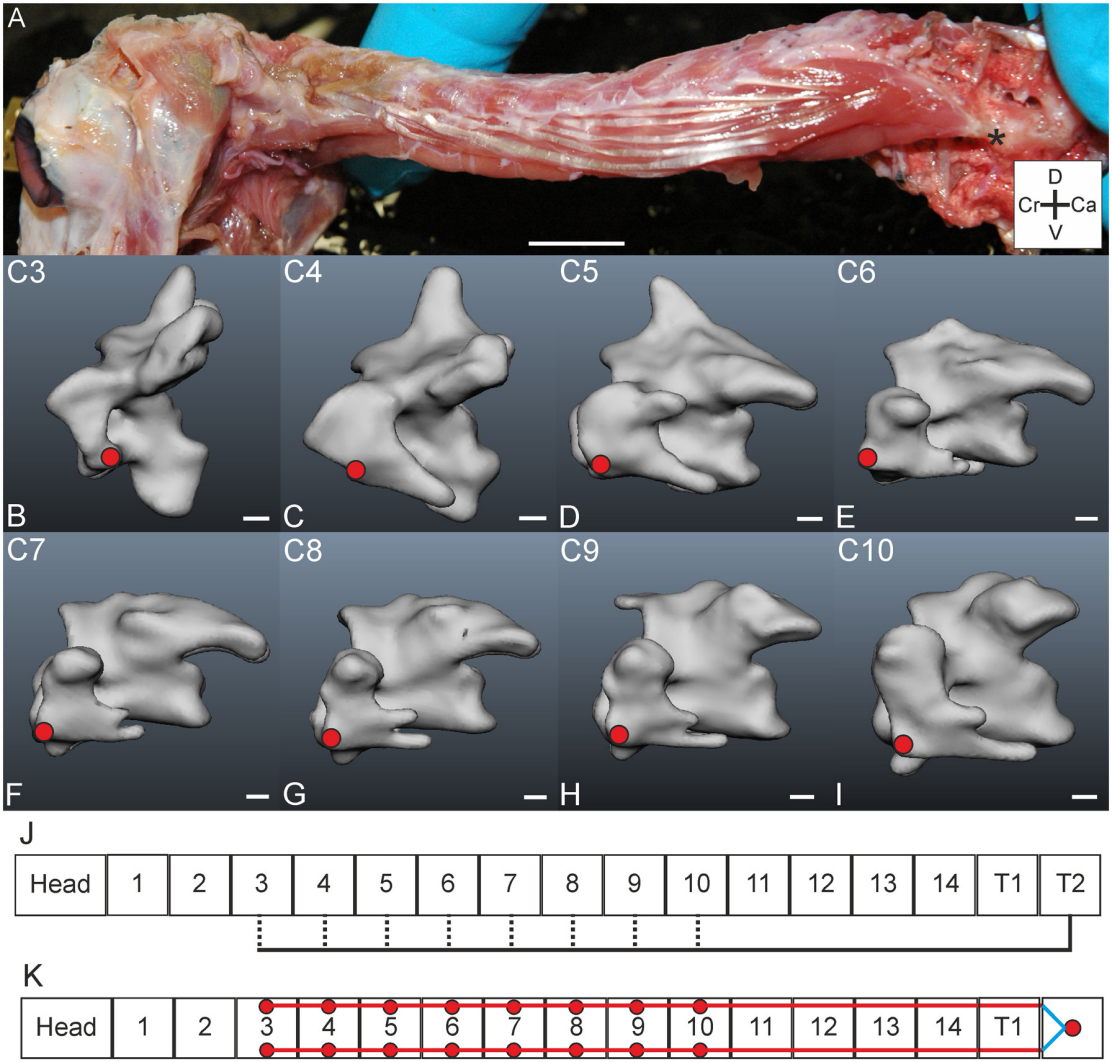
M. longus colli ventralis. A) Lateral view on M. longus colli ventralis. The muscle originates from T2 as indicated by an asterisk. Muscle starts fleshy and becomes aponeurotic and splits when it runs cranially. The aponeurotic parts can clearly be seen in the middle region. Coordinate system indicates dorsal (D), caudal (Ca), ventral (V) and cranial (Cr). Scale bar represents one centimetre. B-I) Muscle attachment sites of the M. longus colli ventralis indicated with red circles in the three-dimensional models of the vertebrae of *T*. *f*. *pratincola*: lateral left view (cranial is to the left). Scale bars represent one millimetre (adapted from [5]). J) Connection diagram from lateral view of M. longus colli ventralis in *T*. *f*. *pratincola*; origin and insertion sites are connected with lines representing the muscle slips, broken lines represent aponeurotic parts. K) Connection diagram from dorsal view of M. rectus capitis ventralis in which the muscle attachment sites are indicated with red circles and are interconnected by lines representing the muscle slips. The blue lines represent ventrally located slips, which are thus actually behind the field of vision in a dorsal view.

*Muscle characteristics*: The M. longus colli ventralis (Fig 12) originates from the processus ventralis of a caudally located vertebra, but not from cervical vertebrae. The bounding fascia was removed to expose individual muscle slips. This muscle first forms a fleshy mass, consisting of both the left and right M. longus colli ventrales. Then the fleshy mass splits into two parts from which each part travels to a side and runs cranially. Finally, eight aponeurotic slips originate on each side from the fleshy mass and run in parallel in a broad overlapping bundle (Fig 12A). From vertebra C10 until C3, one slip sheds off at each vertebra, and inserts at a lateroventral point on the processus transversus (Fig 12B–12I). The tough and complex fascia interferes with a proper dissection of the Mm. cervicales ventrales in *T*. *f*. *pratincola*. Even when the fascia was incised it was hard to separate the individual muscle slips.

*Origin*: In *T*. *f*. *pratincola* the M. longus colli ventralis originates from the processus ventralis of a thoracic vertebra, probably from the second thoracic vertebra (T2) (Fig 12A). This single processus gives rise to both the M. longus colli ventralis sinister and dexter.

*Insertion*: Insertions were found on the processus transversus of C3-C10 (Fig 12B–12I).

*Comparison*: In the mallard (*Anas platyrhynchos*) a pars cranialis and pars caudalis was described and its general morphology is complex [22]. This may indicate that more deeply located slips and attachment sites in *T*. *f*. *pratincola* may have remained unnoticed, although we searched for them. The M. longis colli ventralis as described here for *T*. *f*. *pratincola* may correspond to the pars caudalis in the mallard. According to Baumel et al. (1993), the M. longus colli ventralis inserts to the crista ventralis corporis of the most caudally located cervical vertebrae and to the processus caroticus of intermediate located vertebrae [6].

*Specific discussion*: The M. longus colli ventralis has a ventral path and insertion sites, and an antagonistic function with the M. longus colli dorsalis [21]. From this muscle a functioning in downward rotation of the vertebrae is reported in the black skimmer (*Rynchops nigra*) [16].

### Overview

**Fig 13:**
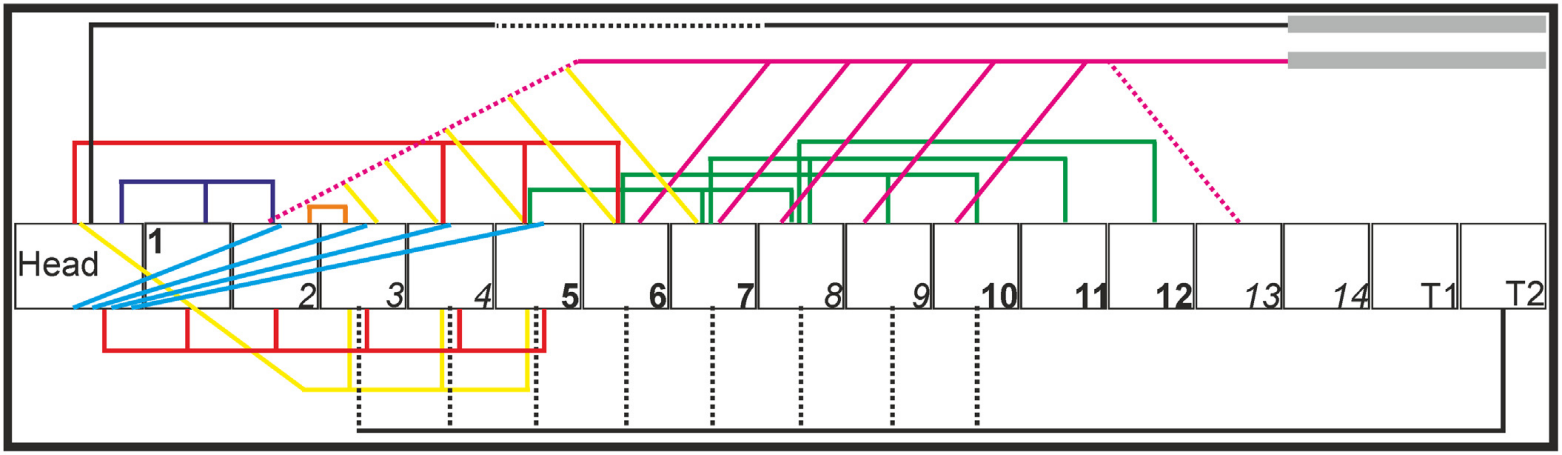
Connection diagram of the cervical muscles as identified in *T*. *f*. *pratincola* from lateral view. The head is represented as a rectangle and the fourteen cervical vertebrae and the first two thoracic vertebrae are represented as squares. The cervical vertebrae are numbered, and the consecutive numbers of the same region as defined by Krings et al. (2014) [5] are represented by the alternating use of bold and italic numbers. Osteological regions were defined as follows; region 1: C1, region 2: C2-C4, region 3: C5-C7, region 4: C8-C9, region 5: C10-C12 and region 6: C13-C14 [5]. The thoracic vertebrae were excluded from the regionalization [5]. Fleshy parts are indicated with solid lines; broken lines represent tendinous or aponeurotic parts. The heavy lines above C14, T1 and T2 represents the aponeurosis notarii. Colours are given for clarity and represent the individual muscles as listed below. Dorsally originating muscles: M. complexus (red), M. biventer cervicis (black), M. splenius capitis (purple), M. rectus capitis dorsalis (blue), M. longus colli dorsalis, pars caudalis (pink), M. longus colli dorsalis, pars cranialis (yellow), pars profunda (green), M. interspinalis (orange). Ventrally originating muscles: M. rectus capitis lateralis (yellow), M. rectus capitis ventralis (red), M. longus colli ventralis (black). Note that this figure represents an overview of the relative muscle positions. This figure does not represent precise attachment sites. These were already provided in Figs 1–12.

**Fig 14:**
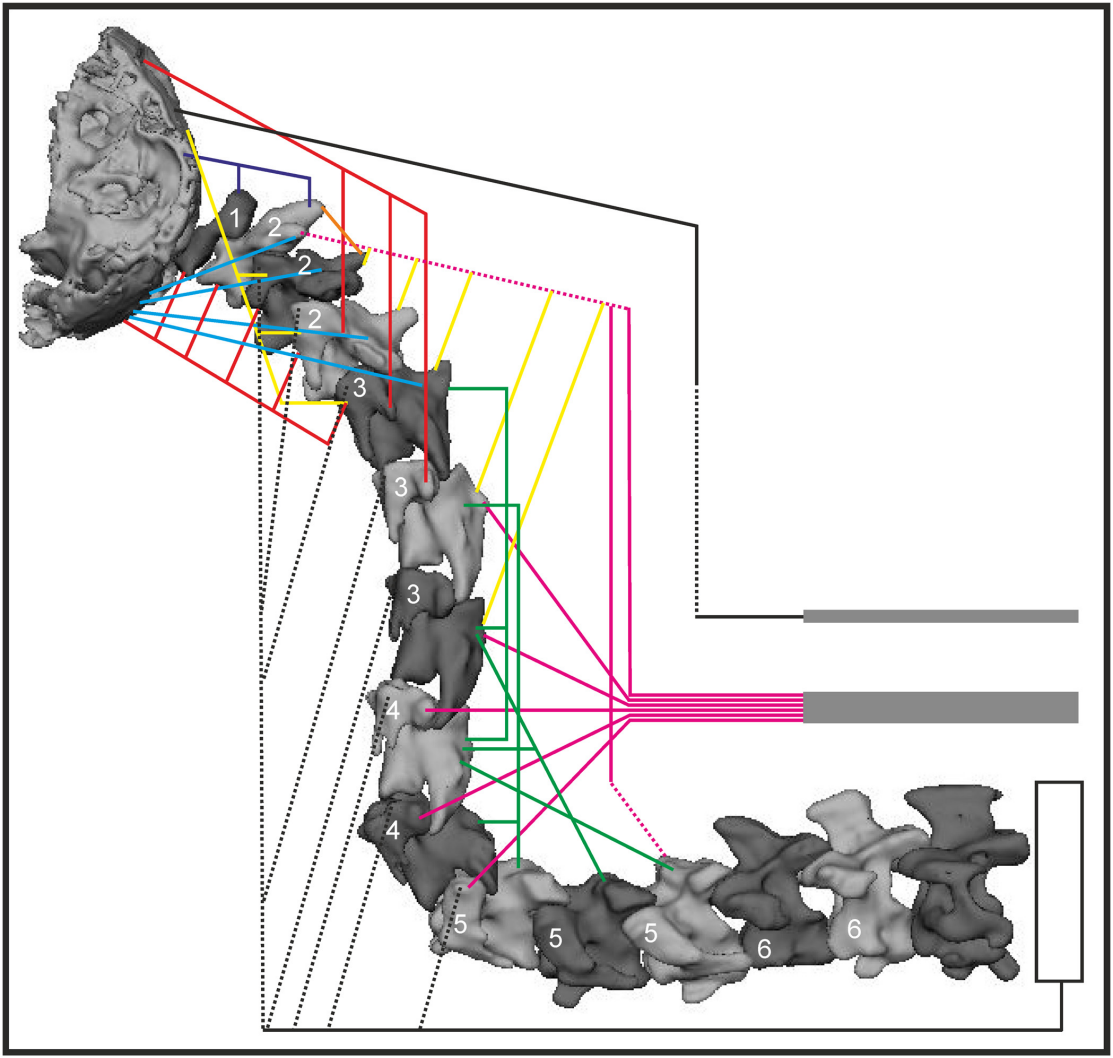
Myology of *T*. *f*. *pratincola* indicated in a semi-diagrammatic reconstruction of the S-shaped neck. Reconstruction of the back of the skull, the 14 cervical vertebrae and the first thoracic vertebra, the second thoracic vertebra is represented diagrammatically, because a three-dimensional model was lacking. The vertebrae are positioned in a natural S-shape which is based on X-ray images made from *T*. *f*. *pratincola* in rest [5]. The numbers in the cervical vertebrae represent the osteological regions as defined by Krings et al. (2014) [5]. Osteological regions were defined as follows; region 1: C1, region 2: C2-C4, region 3: C5-C7, region 4: C8-C9, region 5: C10-C12 and region 6: C13-C14 [5]. Fleshy parts are indicated with solid lines, broken lines represent tendinous or aponeurotic parts. The heavy lines above C14, T1 and T2 represent the aponeurosis notarii. Colours are given for clarity and represent the individual muscles as listed below. Dorsally originating muscles: M. complexus (red), M. biventer cervicis (black), M. splenius capitis (purple), M. rectus capitis dorsalis (blue), M. longus colli dorsalis, pars caudalis (pink), M. longus colli dorsalis, pars cranialis (yellow), pars profunda (green), M. interspinalis (orange). Ventrally originating muscles: M. rectus capitis lateralis (yellow), M. rectus capitis ventralis (red), M. longus colli ventralis (black). Note that this figure represents an overview of the relative muscle positions. This figure does not represent precise attachment sites. These were already provided in Figs 1–12.

As a summary all muscles identified in *T*. *f*. *pratincola* are illustrated in a connection diagram (Fig 13) with the indicated osteological regions [5]. In addition, the identified muscles are illustrated in an S-shaped neck in Fig 14, which makes it easier to interpret the muscle function. Fig 14 also includes the data of regionalization [5]. All muscles of the Mm. craniocervicales reported in literature [6] were identified, and this gives us a complete view how the head may interact with the trunk by muscular contraction. With the description of the M. longus colli dorsalis the major part of the Mm. cervicales dorsales is described. Though some slips of the Mm. cervicales ventrales were described here, this subsystem shows more complexity than described here due to deeper located muscles. The Mm. cervicales laterales were not selected for this study, because they serially repeat, and are complex and deep.

## General Discussion

Because we have already discussed specific issues (see Results and muscle specific discussion), this general discussion will compare the strengths and weaknesses of different methodological approaches, relate myology to head movement capability, compare our earlier results on osteology [5] with the results presented here on myology, and provide a brief outlook to possible future research.

### Methodology

Gross dissection has proven to be a solid and reliable method [2,7,11,13–22] for characterizing avian neck muscles. Previous studies (especially [11,22]) were useful as a reference guide during dissections made here. To our best knowledge the myological description provided in this study is the most detailed documentation of the cervical muscles in a strigiform species presented so far. The data presented here may, therefore, serve as a useful reference guide for further anatomical research on the craniocervical region in birds. However, gross dissection proved to be too challenging for a description of the smallest muscles of *T*. *f*. *pratincola*. Muscles can be stained according to Bock & Shear (1972) to improve the contrast between muscles and other tissue to facilitate the dissection [36], but the determination of the exact muscle attachment site will remain challenging for the minute muscles. The use of contrast-enhanced micro computed tomography is promising [37], and in combination with an iodine staining a useful method for digital dissection of the cervical muscles [10]. We focused on the larger and more superficial muscles, because we assumed that these muscles provide the greater part of the force needed for head movements, in which we are interested. In addition, the longer cervical muscles in birds may contract in the range of centimetres [8], causing substantial movements.

### Adaptive value of myological variation

Because modern birds are the only surviving saurischian dinosaurs, palaeontologists study their morphology as they are related to extinct theropods and sauropods [32,38]. Interspecific variation in myology is common and expressed in variation in size, number of slips and vertebrae of attachment [21]. The variation may be explained as an adaptation to foraging and feeding behaviour [39]. However, also intraspecific variation and even variation within an individual (between the body sides) is a regularly documented phenomenon [21]. Sexual dimorphism was documented for the cervical myology in the huia (*Heteralocha acutirostris*) [27]. Therefore, the greatest care must be taken when drawing conclusions based solely on an anatomical description. Fisher and Goodman (1955) noticed that the variation in muscle arrangement between the body sides (sinister vs. dexter) was correlated with the position of the trachea in the whooping crane (*Grus americana*) [15]. Lateralisation of the trachea was also observed in *T*. *f*. *pratincola* (unpublished observation by the authors). Because lateralisation is not restricted to myology it may even be expressed in behaviour: the lateralisation of the trachea suggests that the characteristic neck flexibility of owls may also be lateralised. The extraordinary head movements as observed in owls require anatomical adaptations [4], but whether specific neck muscles are adapted for this goal remains unclear. Boas’ observation that the M. longus colli dorsalis, pars profunda is more developed in owls than in any other bird he examined [11] hints that these minor muscles may play an important role in owl neck mobility. Recording of an electromyogram of these deeply located and overlapping thin muscles would be illustrative, but may be difficult to obtain. The use of larger species within this order (e.g., *Bubo* spp.) as research material may only partly overcome this problem.

### Myology and head movement capability

The arrangement and architecture of the muscles is the basis for understanding their biological role [40]. It was suggested that head movement patterns are reflected in the cervical morphology [41]. The maximum flexibility of the neck increases after the removal of cervical muscles [3]. This indicates that the muscles do not only (actively) generate the movement of the head, but also (passively) interfere with it. The neck functions to position the head [42]. The avian neck is a redundant system: several combinations of vertebral movements can result in a specific head trajectory [43]. The morphology as observed in *T*. *f*. *pratincola* in which the head is supported by a more or less vertically oriented cervical column may be the result of cost (energy) saving adaptations such as ligaments and osseous processes [44]. The action of individual muscles can be derived relatively easy by imagining what occurs, if the place of insertion is pulled to the place of origin by shortening of the muscle. However, the synergistic contraction of muscles may lead to other functions [16].

The integration of all muscles into one diagram (Fig 13, see also Table 3) demonstrates a two component system underlying the head rotations in barn owls (see also [6]). The first component is composed of the Mm. craniocervicales and serves to move the head relative to the neck. We call this component the “head system”. The head system underlies rotational movements for gaze shifts [32] (analogous to eye movements in humans), as eye movements are restricted to a few degrees in owls [45]. Indeed, the maximal rotational velocities of the head in the barn owl and of the eyes in humans are similar [46,47]. The second component is composed of the Mm. cervicales dorsales, laterales and ventrales. We call this component the “head-neck system”. The head-neck system moves the head with the neck. The head may be regarded as fixed to the neck in these movements. The head-neck system underlies both translational and rotational movements. It is thus similar to the system that causes head movements in humans. However, due to the increased number of cervical vertebrae in birds, the avian equivalent is far more complex.

The head system covers the region where the largest rotational capability is located (Krings et al., in preparation). The M. complexus (dorsal red lines in Figs 13 and 14) inserts dorsally, and may thus be regarded as the antagonist to M. rectus capitis ventralis (ventral red lines in Figs 13 and 14). The position of these muscles suggests that they are involved in dorso- and ventroflexion respectively, as mentioned before. The M. splenius capitis (dorsal blue lines in Figs 13 and 14), the M. rectus capitis lateralis (ventral yellow lines in Figs 13 and 14), and the M. rectus capitis dorsalis (light blue lines in Fig 11) all have lateral and partly dorsoventral paths. These arrangements suggest that these muscles, in addition to dorso- and ventroflexion, add many possibilities for lateroflexion and for rotations about the vertical axis (yaw), and the longitudinal axis (roll). More detailed studies, best including electrical stimuli as in Snively et al. (2014) [32], are necessary to work out the exact roles of the muscles in head rotation. This needs also to include the M. biventer cervicis (dorsal black line in Figs 13 and 14), which, because of its direct connection from the thorax to the head may be involved in a multitude of movements. Masino and Knudsen (1990) mention that the neural circuitry underlying of head movements of *T*. *f*. *pratincola* consists of at least 31 distinct muscle pairs [12]. How these circuits relate to the myology described here and how these units elicit specific head movements remains unclear, however. It should be mentioned that large rotations in particular are not only due to activity in the head system, but also involve the head-neck system, because pure rotations about the vertical axis were not observed in the barn owl [26], and because photographs demonstrated that after 180 degrees of rotation the head is not positioned in the middle of the trunk, but moved sideways (unpublished observation by the authors). To preserve the medulla spinalis, large rotations between vertebrae should be prevented [42]. The head-neck system is composed of several muscles, all of which have the function to position the head-neck complex relative to the trunk. There are two important functions of this positioning: first, to flex the neck and thus position the skull with the eyes and ears so that prey may be fixated. This includes both translational and rotational movements. Second, this system may impose roll movements on the vertebrae from about C10 to C14. In general, dorsal muscles are used for dorsal flexion and retraction, ventral muscles for ventral flexion and lateral muscles cause lateral flexion and rotation [22]. This roll movement is crucial for the large head movement capability of the American barn owl ([5], Krings et al., in preparation).

### Comparison of neck osteology with neck myology

Previously presented osteological data on the cervical vertebrae of *T*. *f*. *pratincola* [5] suggested a subdivision of the owl’s neck into seven regions [5]. By contrast, myology divides the neck muscles into four regions [6] that we divided into two functional components. This already suggests incongruence between overall myology and osteology with the current criteria. The origins of the Mm. craniocervicales extend from C1 to C6 (excluding the M. biventer cervicis which originates from the aponeurosis notarii above C14). This corresponds to region one to three in osteology. Specifically, there was a border in osteology between C4 and C5, while slips of the same muscle (M. complexus) attach to both C4 and C5. Similar observations hold for the region from C10 to C12, where the M. longus colli dorsalis, pars profunda originates from all vertebrae, but also from C7-C9 which belongs to other osteological regions [5]. While it is possible that the neuromotor units allow for a separate activation of each muscle slip, the myology as described here clearly does not correlate well with the regionalisation as found in osteology. On the other hand, osteology and myology are closely related because the load of a muscle determines the morphology of a bone (Wolff’s law) [48], and the size of a muscle attachment site reveals the strength of a muscle: relatively small attachment sites suggest less forceful muscles [25]. We suggest that the M. complexus, M. splenius capitis, M. rectus capitis lateralis, M. rectus capitis ventralis, M. rectus capitis dorsalis are responsible for the large rotations in the most cranial region of the vertebral column, from which the highest degrees of rotation may be realised by the articulatio atlanto occipitale and articulatio atlantoaxialis.

### Possible future directions

The cervical anatomy of *T*. *f*. *pratincola* has not yet been worked out completely. What is still missing is an account of the small and deeper located muscles (e.g., Mm. cervicales ascendentes and Mm. intertransversarii). A description of these muscles will further elucidate how owls pivot their heads. The Mm. cervicales ascendentes are well developed in the golden eagle (*Aquila chrysaetos*) [25], and unilateral contraction may cause interesting vertebral rotation in owls. Additionally the effect of ligaments, and the role of the intervertebral cartilage remained undescribed, but may all influence neck flexibility [3]. For example, the thickness of intervertebral cartilage highly influences neck flexibility [49] and data on this would enable us to fully integrate the anatomical data to model the head movements of *T*. *f*. *pratincola*. The physiological cross sectional area may give a clue about the force achievements of the cervical musculature. Moreover, physiological approaches like electromyography studies are needed to find out how the muscles act in concert enabling the extreme head movements of owls.
